# The *Drosophila* TIPE family member Sigmar interacts with the Ste20-like kinase Misshapen and modulates JNK signaling, cytoskeletal remodeling and autophagy

**DOI:** 10.1242/bio.20148417

**Published:** 2015-04-02

**Authors:** Suganthi Chittaranjan, Jing Xu, Michael Kuzyk, Harpreet K. Dullat, James Wilton, Lindsay DeVorkin, Chandra Lebovitz, Gregg B. Morin, Marco A. Marra, Sharon M. Gorski

**Affiliations:** 1 The Genome Sciences Centre, BC Cancer Agency, 675 West 10^th^ Avenue, Vancouver, BC V5Z 1L3, Canada; 2 Department of Molecular Biology and Biochemistry, Simon Fraser University, Burnaby, BC V5A 1S6, Canada; 3 Department of Medical Genetics, The University of British Columbia, Vancouver, BC V6T 1Z3, Canada

**Keywords:** Eiger, Msn, Autophagy, Cytoskeleton, TNFAIP8, TIPE

## Abstract

TNFAIP8 and other mammalian TIPE family proteins have attracted increased interest due to their associations with disease-related processes including oncogenic transformation, metastasis, and inflammation. The molecular and cellular functions of TIPE family proteins are still not well understood. Here we report the molecular and genetic characterization of the *Drosophila* TNFAIP8 homolog, *CG4091/sigmar*. Previous gene expression studies revealed dynamic expression of *sigmar* in larval salivary glands prior to histolysis. Here we demonstrate that in *sigmar* loss-of-function mutants, the salivary glands are morphologically abnormal with defects in the tubulin network and decreased autophagic flux. Sigmar localizes subcellularly to microtubule-containing projections in *Drosophila* S2 cells, and co-immunoprecipitates with the Ste20-like kinase Misshapen, a regulator of the JNK pathway. Further, the Drosophila TNF ligand Eiger can induce *sigmar* expression, and *sigmar* loss-of-function leads to altered localization of pDJNK in salivary glands. Together, these findings link Sigmar to the JNK pathway, cytoskeletal remodeling and autophagy activity during salivary gland development, and provide new insights into TIPE family member function.

## Introduction

Human Tumor Necrosis Factor-alpha Induced Protein 8 (TNFAIP8)/SCC-S2/NDED/GG2-1 was first identified in a differential display screen that identified tumor necrosis factor-α (TNF-α) responsive genes in endothelial cells (Horrevoets et al., 1999) and was later implicated as a regulator of apoptosis as well as other processes relevant to human disease (Kumar et al., 2000; Kumar et al., 2004; Laliberté et al., 2010; Woodward et al., 2010; Zhang et al., 2010). In MDA-MB-435 breast cancer cells, exogenous expression of TNFAIP8 caused enhanced DNA synthesis, cell proliferation, and tumor growth rate (Kumar et al., 2004) and promoted invasion (Zhang et al., 2006). In patients with non-small-cell lung cancer, over-expression of TNFAIP8 correlated with lymph node metastasis and poor prognosis (Dong et al., 2010). TNFAIP8 was also shown to interact directly with the Gα(i) receptor to enhance progression to oncogenic transformation (Laliberté et al., 2010). In patients with epithelial ovarian cancer, TNFAIP8 predicted metastasis and poor survival (Liu et al., 2013). These observations and others (Woodward et al., 2010; Zhang et al., 2010) have led to the proposal that TNFAIP8 may be a useful therapeutic target for human disease. However, the molecular and cellular mechanisms associated with TNFAIP8 activity are still not well understood, and the roles of TNFAIP8 in the context of normal development are not yet well characterized.

TNFAIP8 is the founding member of a recently described TNFAIP8-like (TIPE) protein family. Based on sequence similarity, another three mammalian TIPE family members may exist and are designated TIPE1 (TNFAIP8-like 1), TIPE2 and TIPE3 (Luan et al., 2013; Sun et al., 2008).TIPE2 mRNA is also induced by TNF-α and TIPE2 was shown to function as a negative regulator of immune cell function, acting to prevent hyper-responsiveness and inflammation, and thus maintain immune homeostasis (Luan et al., 2013; Sun et al., 2008). TNFAIP8 homologs have been identified in several mammals (e.g. human, mouse, rat, dog) and in chicken, zebrafish, mosquito and the fruit fly, *Drosophila melanogaster* (Sayers et al., 2011). Differential expression of the *Drosophila* homolog, *CG4091*, was associated with salivary gland histolysis (Gorski et al., 2003; Lee et al., 2003) but no functional studies of *CG4091* have been reported.

During metamorphosis of *Drosophila*, a pulse of the steroid hormone 20-hydroxyecdysone (20HE) at 10–12 hours After Puparium Formation (APF; at 25°C) triggers cell death of the larval salivary glands (Jiang et al., 1997). This ecdysone pulse initiates a transcriptional cascade in the larval salivary glands involving multiple ecdysone-response genes and apoptosis genes. A detailed description of the genes involved and their hierarchy in histolysing larval salivary glands can be found in several reports (Baehrecke, 2000; Baehrecke, 2003; Gorski et al., 2003; Lee et al., 2000; Lee et al., 2003; Yin and Thummel, 2005). During death of *Drosophila* larval salivary glands, extensive rearrangement and depolymerization of the cytoskeletal network has been observed. Two comprehensive studies on the changes in the cytoskeletal arrangements during salivary gland cell death showed reorganization of actin filaments and rearrangement and eventual depolymerization and disintegration of tubulin and nuclear lamins (Jochová et al., 1997; Martin and Baehrecke, 2004). Complete *Drosophila* salivary gland cell degradation also requires the process of autophagy (Berry and Baehrecke, 2007), an intracellular catabolic pathway for the degradation and recycling of cytoplasmic components and organelles. Autophagy is frequently associated with cell survival (Juhász et al., 2007; Qadir et al., 2008) but also with tissue and cell degradation (Berry and Baehrecke, 2007; Hou et al., 2008), and is characterized by the formation of double-membrane autophagosomes that engulf cytoplasmic constituents. The autophagosomes subsequently fuse with lysosomes to form autolysosomes where degradation occurs. Autophagy has been implicated in differentiation, morphogenesis and other developmental events in both invertebrates and vertebrates (Meléndez and Neufeld, 2008).

In this study, we developed a genetic model of *sigmar*, the *Drosophila* TIPE family member. Gene expression of *sigmar* is increased dramatically in *Drosophila* larval salivary glands prior to histolysis, and we found that in *Drosophila*, similar to mammals, *sigmar* expression can be further increased in response to the TNF ligand Eiger. In *Drosophila* S2 cells, Sigmar protein localizes to discrete patches on cellular extensions in a microtubule-dependent manner, and Sigmar co-immunoprecipitates with cytoskeletal proteins and the JNK pathway regulator Misshapen (Msn). Analyses of sigmar loss-of-function in *Drosophila* salivary glands demonstrate a functional requirement in the JNK signaling pathway, cytoskeleton organization and autophagy during salivary gland development.

## Results

### Creation and analysis of a null mutant in the *Drosophila* TIPE family member *sigmar*

**Fig. 1.**
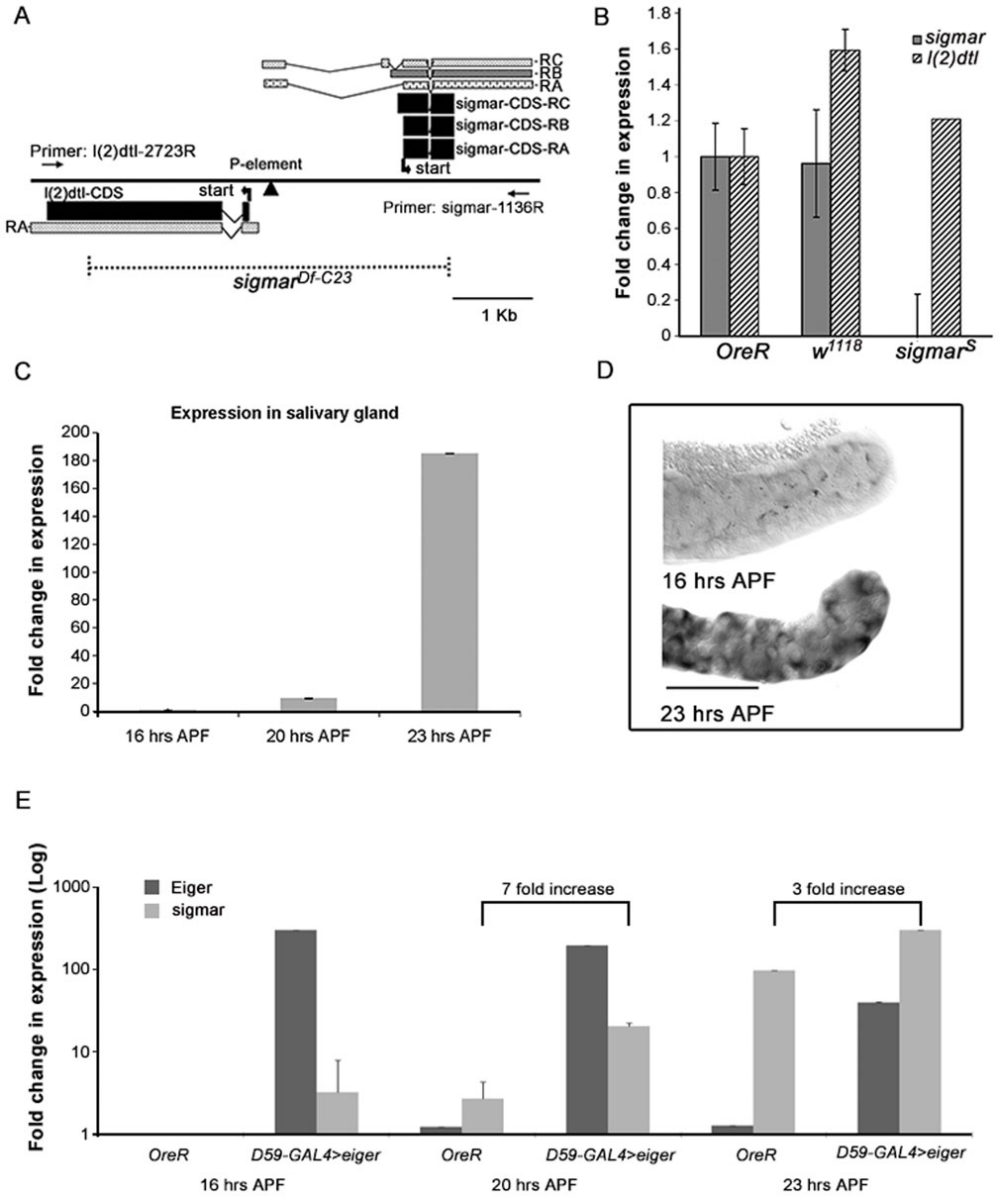
Creation and analysis of a genetic model of the Drosophila TIPE family member *sigmar*. (A) The *sigmar* gene has three transcripts (RA, RB, RC) that encode two different proteins. Sigmar-CDS-RA and Sigmar-CDS-RB encode the same protein (188 aa) and Sigmar-CDS-RC encodes a protein that is slightly larger (210 aa). The *sigmar* and *l(2)dtl* genes are separated by a 78 bp region. The strain EY06821 contained a P element inserted in the 3′UTR region of *sigmar* (arrowhead). Broken lines indicate the genomic region deleted in the *sigmar^Df-C23^* strain generated by the imprecise excision of EY06821. Primers l(2)dtl 2723R and Sigmar 1136R were used to detect the deletion PCR product of *sigmar^Df-C23^*. The *sigmar^Df-C23^* strain has a deletion in both *l(2)dtl* and *sigmar* gene coding regions. (B) QRT-PCR analysis indicates that the *sigmar^S^* strain does not express *sigmar* but does express *l(2)dtl* transcripts similar to the control *OreR* and *w^1118^* strain. Fold expression shown (mean ± s.d.) is relative to expression levels in *OreR* females using rp49 as the reference RNA. (C) QRT-PCR results show that *sigmar* expression increased dramatically (approximately 185 fold) prior to the death stage (23 hours APF at 18°C) in *OreR* larval salivary glands. Fold change in expression (mean±s.d.) is relative to the 16 hours APF time point using rp49 as the reference RNA. (D) *In situ* hybridization of larval salivary glands confirms that *sigmar* expression is not detectable at 16 hours APF at 18°C but is abundant at the death initiating stage, 23 hours APF. Scale bar equals 100 microns. (E) *sigmar* expression is increased in *eiger*-expresssing salivary glands. Salivary glands were dissected at 16 hours APF, 20 hours APF and 23 hours APF (at 18°C) from wild-type (*OreR*) animals and from a strain ectopically expressing *eiger* in salivary glands (*D59-Gal4>eiger*). The mRNA expression levels of *eiger* and *sigmar* were determined using QRT-PCR and are shown as fold changes in expression (Log scale, mean±s.d.) relative to *OreR* at 16 hours APF. Results show that *sigmar* expression was increased 7 fold and 3 fold in the *eiger*-expressing salivary glands relative to wild type at 20 hours APF and 23 hours APF, respectively.

Based on both DNA and amino acid sequence comparisons, the *Drosophila* genome contains a single TIPE family member (gene id CG4091) with an amino acid sequence most similar to human TNFAIP8. We named this *Drosophila* gene *sigmar* (salivary glands marred), based on its loss-of-function phenotype described below. TNFAIP8 and Sigmar show 42% amino acid identity and 65% amino acid similarity [based on BLAST analysis (Altschul et al., 1990)] for the 188 aa protein sequence. Sigmar also shares a high amino acid identity (39%) and similarity (62%) with TIPE2, the other characterized TNFAIP8 family member (Sun et al., 2008). To determine the function of *sigmar* during development, we created a null mutant by P-element imprecise excision. Our strategy involved mobilization of a P transposon insertion, EY06821 (Crosby et al., 2007), located in the 5′UTR of the *sigmar* gene (Fig. 1A). A null mutant for Sigmar, *Df(2R)l(2)dtl-sigmar* (hereafter referred to as *sigmar^Df-C23^*), that removes part of the upstream gene *l(2)dtl* (Kurzik-Dumke et al., 1996) and all the *sigmar* 5′UTR and most of the *sigma*r coding sequence was identified by genomic PCR and sequence analyses (Fig. 1A). To create a loss-of-function mutant strain specific for *sigmar*, we created a pUAST-*l(2)dtl*^42-3^ expression strain to rescue *l(2)dtl* in *sigmar^Df-C23^* animals. The resulting strain, *sigmar^Df-C23^/sigmar^Df-C23^*; *pUAST- l(2)dtl^42-3^/TM6B* (hereafter referred to as *sigmar^S^*) was viable and developed to adulthood with basal level *l(2)dtl* expression without any GAL4 drivers. We confirmed the presence of *l(2)dtl* expression and the absence of *sigmar* expression in this mutant strain by QRT-PCR (Fig. 1B). We also created a control rescue strain for both *l(2)dtl* and *sigmar* genes: *sigmar^Df-C23^/sigmar^Df-C23^*; *pUAST-l(2)dtl^42-3^/pUAST-sigmar^15-1^* (hereafter referred to as *sigmar^res^*). Both *sigmar^S^* and *sigmar^res^* developed to adult stages similar to wild-type *w^1118^* with no obvious morphological abnormalities.

### *sigmar* transcripts increase in larval salivary glands prior to histolysis and can be induced in response to Eiger, the *Drosophila* TNF-ligand

Two previous large-scale gene expression studies indicated that *Drosophila sigmar* showed significant differential expression in salivary glands, with dramatically increased expression just prior to developmental cell death (Gorski et al., 2003; Lee et al., 2003). Given the absence of any overt developmental defects in *sigmar^S^* mutants, we chose to further utilize this tissue to investigate the potential functional roles of Sigmar during development *in vivo*. First, to independently validate and to quantitate the expression levels of *sigmar* in larval salivary glands prior to and during developmental cell death, we employed quantitative reverse transcription PCR (QRT-PCR) and measured transcript levels in wild-type *OreR* salivary glands (16, 20 and 23 hours APF at 18°C; equivalent to 8.5, 11 and 12.5 hours APF at 25°C). As demonstrated in Fig. 1C *sigmar* showed elevated abundance by 20 hours APF (9-fold increase) and dramatically increased RNA accumulation at 23 hours APF (185-fold increase in expression) prior to salivary gland histolysis. To verify temporal and spatial expression of *sigmar* in larval salivary glands, we performed *in situ* hybridization to this tissue. In accordance with the QRT-PCR results, *sigmar* RNA was not detected at 16 hours APF but was detected at 23 hours APF (Fig. 1D), correlating with the initiation of larval salivary gland histolysis. These expression studies confirm that *sigmar* is expressed in salivary gland tissue and that its expression level is elevated prior to the onset of salivary gland histolysis. Consistent with FlyAtlas Anatomical Expression Data (Chintapalli et al., 2007), we also observed relatively high expression of *sigmar* in the embryonic brain and mid-gut (supplementary material Fig. S1A–E), and in the larval mid-gut prior to steroid induced cell death (supplementary material Fig. S1F).

Sigmar may be a functional orthologue of TNFAIP8 and participate in a *Drosophila* TNF-like signaling pathway. To determine whether Eiger, the *Drosophila* homolog of TNF-α can induce sigmar gene expression, we ectopically expressed Eiger in *Drosophila* salivary glands using the UAS-Gal4 system (Brand and Perrimon, 1993). QRT-PCR analyses of salivary gland RNA indicated that overexpression of Eiger in salivary glands led to enhanced expression levels (3–7 fold increase) of *sigmar* at three different time points examined (Fig. 1E). These data indicate that, similar to increased expression of TNFAIP8 in response to mammalian TNF-α, the expression of sigmar is enhanced in response to the Drosophila TNF-α homolog Eiger. Together, the evolutionary sequence conservation, null mutant line, developmental regulation, and Eiger-responsiveness make *Drosophila* a useful model for investigating TIPE function.

### Sigmar is associated with cellular projections in S2 cells

**Fig. 2.**
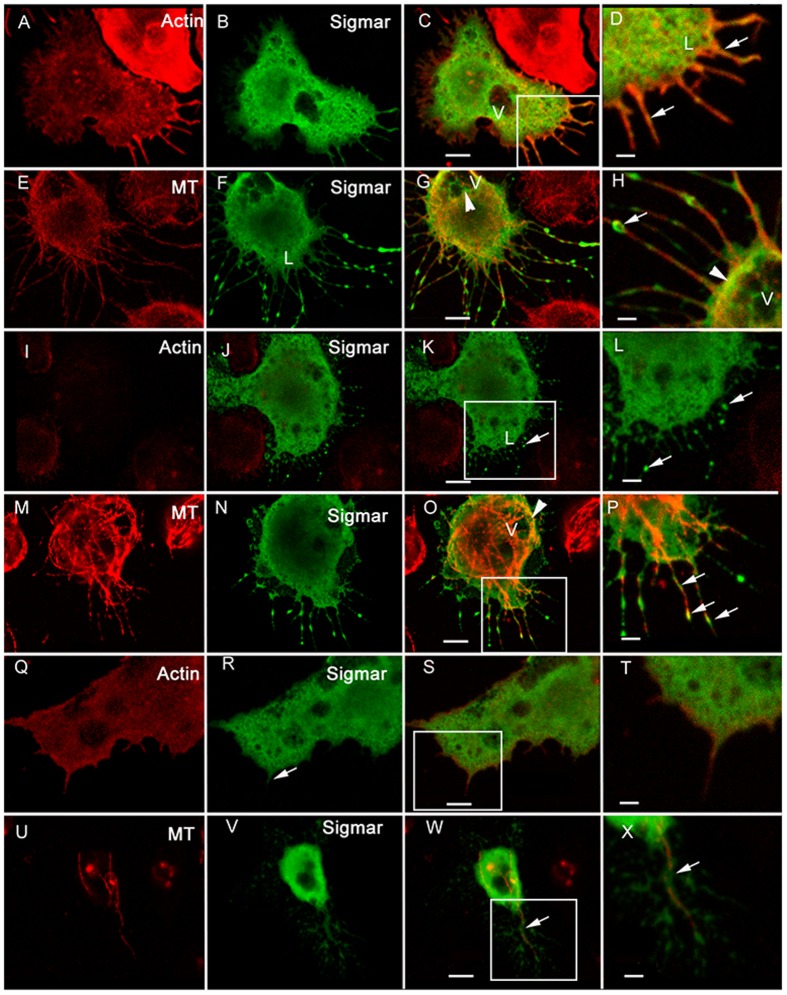
Sigmar localizes to cellular projections in S2 cells growing on CC2 coated slides. A–H show untreated S2 cells, while I–P show S2 cells treated with latrunculin B (actin-disrupting) for 15 minutes and Q–X show cells treated with vinblastine (microtubule-disrupting) for 35 minutes. A cell stained for actin with rhodamine-phalloidin (A, red) and Sigmar (B, green) shows some co-localization of actin and Sigmar (C,D; yellow-orange) in the extended processes (D, arrow); ‘V’ = vacuolar structure. A cell stained for tubulin, a component of microtubules (MTs) (E, red), and Sigmar (F, green) also shows co-localization, especially in the long extended protrusions (G) and around the vacuoles (panel G, labelled ‘V’ and with an arrowhead). Sigmar appears as patches along the protrusions and at the tips (H, arrow). Latrunculin B treatment disrupts actin (I, red), however, Sigmar (J, green) still localizes in the lamellar areas (‘L’) and along the protrusions (panels K and L, arrows). (M–P) A latrunculin B treated cell stained for tubulin (M, red) and Sigmar (N, green) depicts Sigmar protein co-localizing to intact tubulin particularly surrounding vacuoles (O, arrowhead) and in cellular extensions (P, arrows) similar to untreated cells. Disruption of tubulin with vinblastine does not affect the actin filaments (Q, red) and Sigmar (R, green) is still present in the central area of the cells but little Sigmar protein was observed in the extensions (R, arrow). The overlay (S) and magnified view of actin extensions (T) are also shown. Vinblastine treatment disrupted the majority of tubulin filaments (U, red) but some tubulin filament remnants remained (U). Sigmar (V, green) co-localized with these remnants (panel W–X; arrows) but appeared reduced. Scale bars in A–C,E–G,I–K,M–O,Q–S,U–W equal 5 microns. Scale bars in D,H,L,P,T,X equal 25 microns. Boxed areas in C,K,O,S,W show the regions magnified in subsequent panels.

To provide further insights into the function of Sigmar, we determined its subcellular localization in *Drosophila* S2 cells using N-terminus or C-terminus FLAG-tagged Sigmar (F-Sigmar or Sigmar-F, respectively) and an anti-FLAG antibody. With both constructs, we observed a cytoplasmic distribution of Sigmar as well as localization along cellular projections (Fig. 2). To investigate this distribution further, we employed CC2^TM^ coated slides to study the actin and tubulin network under *in vitro* conditions as described previously (supplementary material Fig. S2A,B) (Rogers et al., 2003). At 4–5 hours after plating, most cells contained both short and long projections that stained with phalloidin, indicating that the projections contain actin filaments (Fig. 2A–D; supplementary material Fig. S2C, arrows). The projections also contain tubulin (Fig. 2E). To determine whether Sigmar localizes to actin or microtubules (MTs) in the cells with projections, we performed immunofluorescence (IF) detection using Sigmar (i.e. FLAG), actin (i.e. rhodamine-phalloidin) and tubulin probes. These analyses revealed that Sigmar protein appears to localize with actin in the projections (Fig. 2A–D). Patches of Sigmar protein were also observed along the long tapered projections (Fig. 2E–H) where we also clearly observed robust staining for MTs as detected by anti-tubulin antibody.

To investigate the dependence of Sigmar's localization to projections on actin and/or MTs, we treated cells with latrunculin B, a drug that disrupts actin microfilaments, or vinblastine, a drug that causes depolymerization of MTs. Latrunculin B treatment for 5 to 25 minutes resulted in dramatic disruption of the actin cytoskeleton, which was evident by phalloidin staining (Fig. 2I; supplementary material Fig. S2D,F,H). Following the disruption of actin filaments, a considerable amount of Sigmar staining was still observed, particularly along long tapered projections (Fig. 2J–L; supplementary material Fig. S2D–I). Co-staining for Sigmar and tubulin in these cells confirmed that Sigmar and MTs co-localize in some regions of the remaining long extensions (Fig. 2M–P; supplementary material Fig. S2E,G,I). However, Sigmar often appears as a larger “patch” of staining. Vinblastine treatment for 30 to 40 minutes resulted in disappearance of both the protrusions and long projections and associated Sigmar-Flag staining (Fig. 2Q–T,U–X). A cytoplasmic distribution of both Sigmar and phalloidin was still observed in the vinblastine treated cells (Fig. 2Q–T) in the lamellar region. Where remnants of tubulin filaments persisted, low levels of Sigmar staining could still be observed also (Fig. 2U–X). Together, these observations indicate that Sigmar associates with cellular projections that are dependent on intact MTs and actin.

We also used Drosophila S2 cells and the F-Sigmar and Sigmar-F constructs to identify candidate Sigmar-interacting proteins by immuno-affinity purification (IP) and tandem mass spectrometry (MS/MS). We identified 39 proteins that co-immunoprecipitated with both Sigmar constructs but not the empty vector control (Table 1; supplementary material Table S2; see Materials and Methods for details of criteria used). Consistent with our subcellular localization results, we identified the cytoskeletal structural components αTub84B and Act42A, and microtubule binding proteins Map205 and pav (Table 1) as candidate interaction partners of Sigmar protein. Three kinases, Ste20-like kinase msn, par-1, and polo, known to regulate cytoskeleton dynamics during *Drosophila* development (do Carmo Avides et al., 2001; Doerflinger et al., 2003; Su et al., 1998a) were also identified as candidate Sigmar-interacting proteins (Table 1). These findings further point toward a potential role for Sigmar in cytoskeleton-related processes.

**Table 1.**
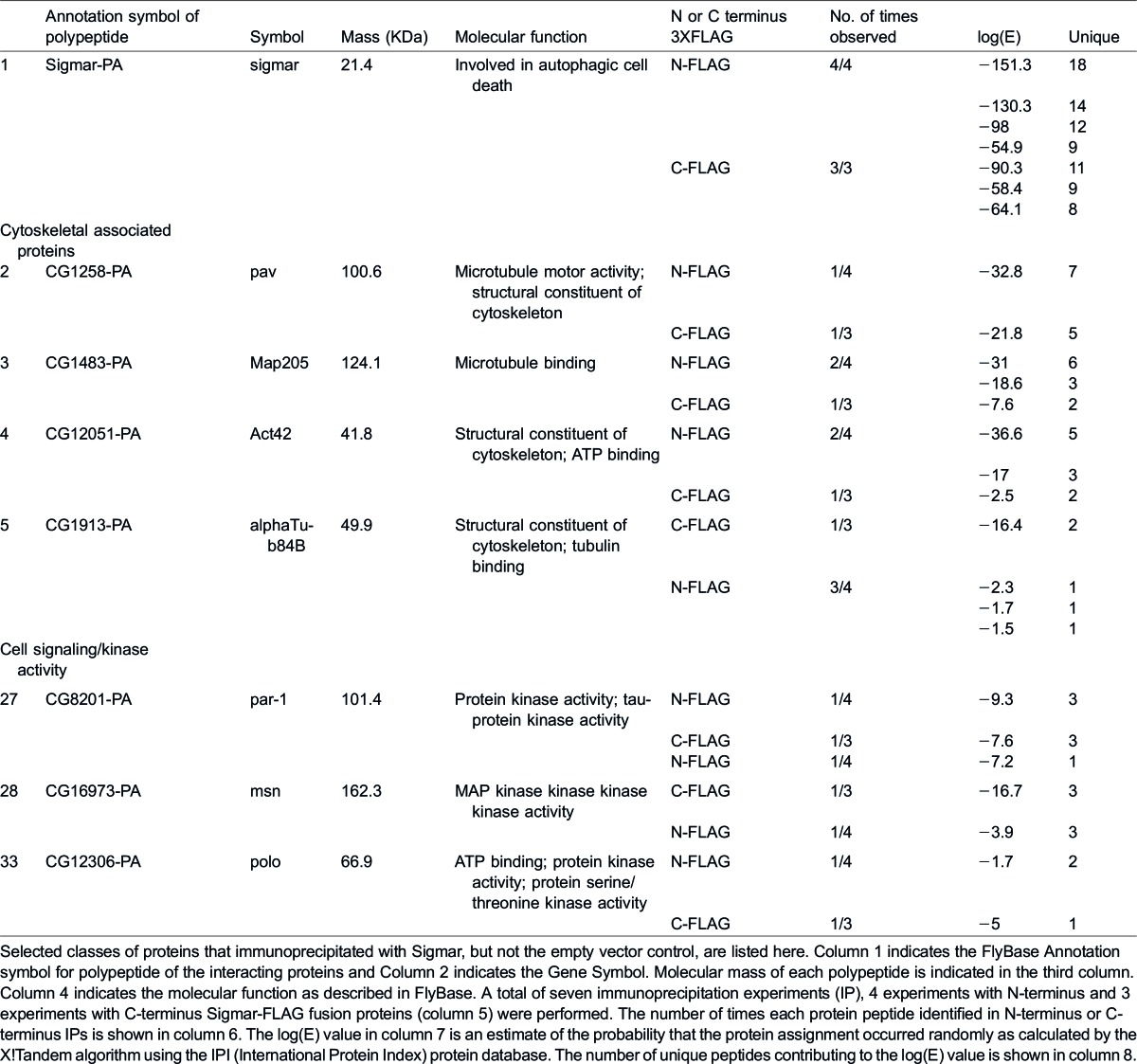
Candidate cytoskeletal and cell signaling related interaction partners of Sigmar

### Sigmar facilitates MT remodeling in salivary glands

**Fig. 3.**
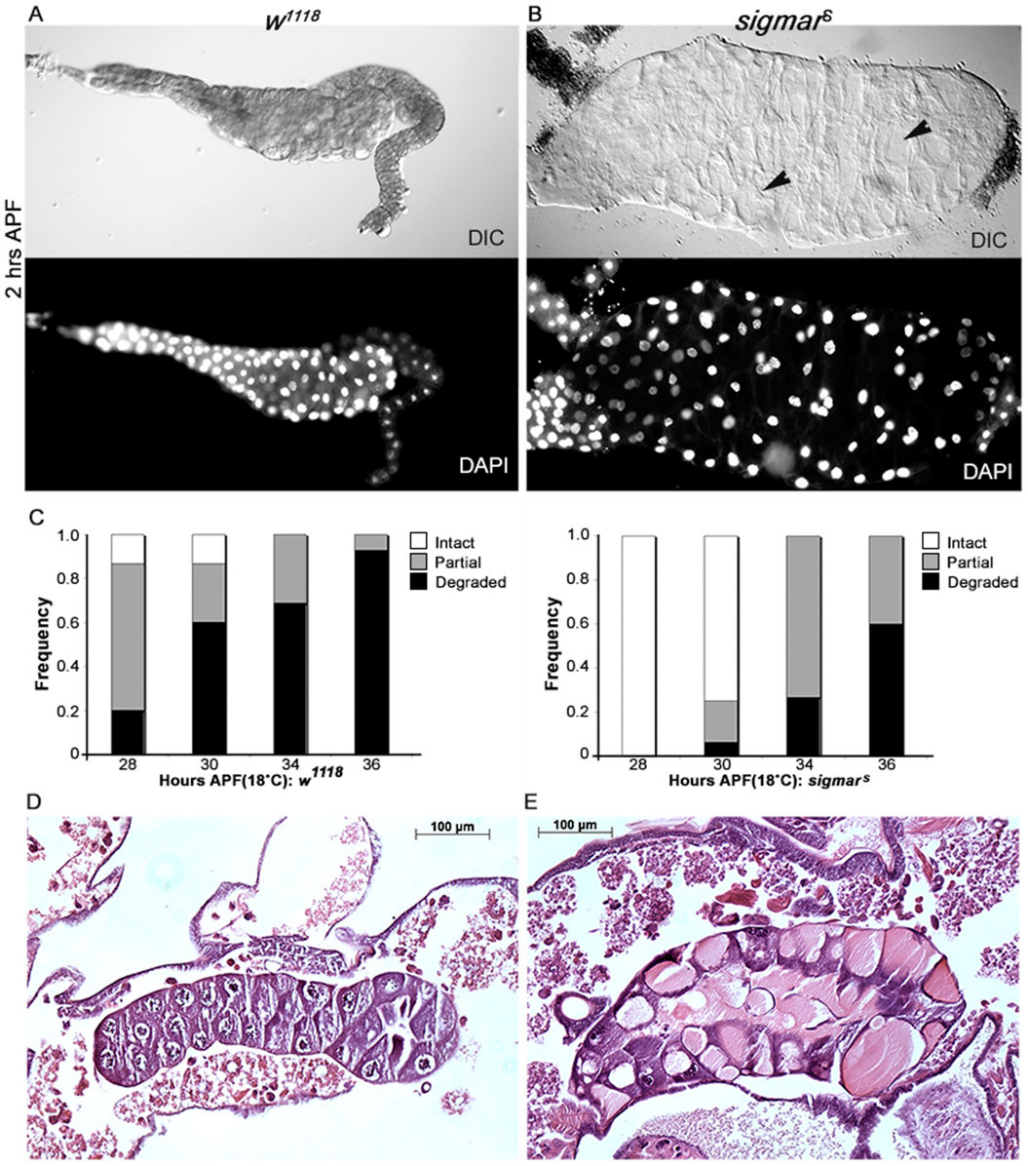
Salivary glands of *sigmar^S^* mutants are morphologically abnormal. (A) Salivary glands from control (*w^1118^*) and (B) *sigmar^S^* null mutant pupa (2 hours APF). Compared to wild-type (*w^1118^*) salivary glands in A, *sigmar^S^* salivary glands in B are comprised of abnormally enlarged cells that appeared to contain large empty vacuoles (black arrows) that were clearly evident after 1–2 hours APF at 18°C. Note also the abnormal arrangement of nuclei (B, DAPI) in the mutant gland at this stage. Images were captured with a Zeiss fluorescence microscope (×10 objective). Differential interference contrast (DIC) images are shown with the corresponding DAPI stained salivary glands below. (C) Salivary gland histolysis is delayed in *sigmar^S^* mutants compared to *w^1118^* controls. White pre-pupae were incubated at 18°C for indicated time period. Salivary glands were harvested and scored for the extent of histolysis (n>10). (D) H&E staining of salivary glands from whole fixed pupae at 24 hours APF in *w^1118^* controls is shown (n = 6). (E) H&E staining of salivary gland thin section from whole fixed pupae 24 hours APF in *sigmar^s^* mutants. Whole salivary glands appear enlarged with vacuole-like structures. H&E staining positive regions are apparent (n = 6).

Given the association of Sigmar with the cytoskeleton and its reported dynamic gene expression in salivary glands, we next investigated whether Sigmar had a role in the cytoskeletal remodeling known to occur during salivary gland development (Jochová et al., 1997). Salivary glands were dissected from control and *sigmar^S^* mutants from time points ranging from the 3rd instar wandering larval stage to salivary gland histolysis in the mid-pupal stage. Gross morphology of all of the *sigmar^S^* mutant salivary glands appeared normal up to the wandering larval stage (compare supplementary material Fig. S4A,B) but exhibited a slight increase in overall length (supplementary material Fig. S4C). In the pupal stages examined, all of the mutant salivary glands were significantly longer and wider (supplementary material Fig. S4C,D) compared to wild-type glands and were morphologically abnormal (compare Fig. 3A,B) with some of the cells appearing grossly enlarged with big empty vacuole-like structures (Fig. 3B). The timing of the first apparent morphological defects (i.e. at 2 hours APF; Fig. 3B) in the salivary glands of *sigmar^S^* coincides with the extensive rearrangement of tubulin and migration of nuclei towards the basal membrane in salivary glands (Jochová et al., 1997). Similar to wild type, the salivary glands from *sigmar^S^* mutants persisted for at least up to 25–26 hours APF (at 18°C). Salivary gland histolysis still occurred in the *sigmar^S^* mutants but appeared delayed compared to control animals (*w^1118^*) (Fig. 3C). Analysis of thin sections from whole fixed pupae at 24 hours APF revealed persistence of the abnormally large vacuole-like phenotype (Fig. 3D,E) until eventual salivary gland destruction.

**Fig. 4.**
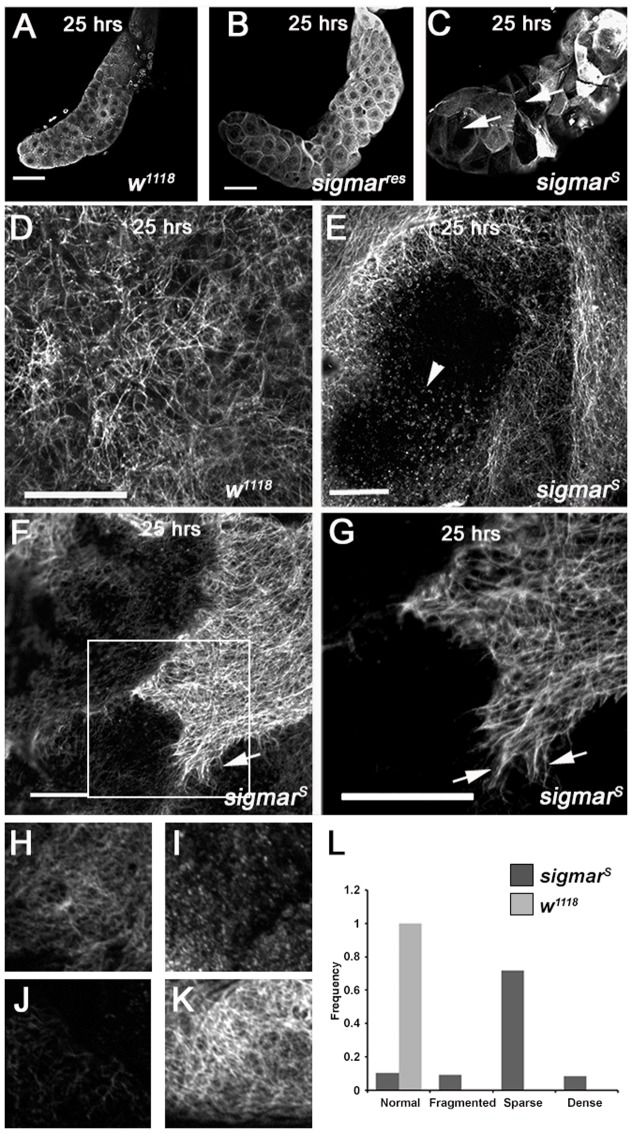
Salivary glands from *sigmar^S^* null mutants show defects in the tubulin network. (A) Salivary glands from *w^1118^* control and (B) *sigmar^res^* strain control (rescue strain for *sigmar^S^*) appear normal. (C) Salivary glands from the *sigmar^S^* null-mutant stained for tubulin using an anti-beta tubulin antibody, appeared abnormal with some grossly enlarged cells (C, arrows). (D) Magnified view of a cell in control animals shows a normal tubulin network. (E) Magnified view of one of the grossly enlarged salivary gland cells from the *sigmar^S^* mutant shows a fragmented tubulin network (E, arrowhead). (F,G) Some cells in the *sigmar^S^* mutant salivary glands displayed a dense tubulin network pattern with long microtubules that appeared as fibers. Arrows in magnified panel G show these long ‘fiber-like’ microtubules. Salivary glands cells from *w^1118^* and *sigmar^S^* were categorized as having a normal (H), fragmented (I), sparse (J) or dense (K) tubulin network and the frequency of each cell phenotype from *w^1118^* (n = 10 glands) and *sigmar^S^* (n = 10 glands) determined. A total of 497 cells were quantified and results presented as a bar graph (L). Scale bars in A–C equal 100 microns, in D–G equal 20 microns. Images A–G were taken with a Nikon confocal microscope and shown is a single Z slice. Images H–K were taken with a Zeiss fluorescence microscope using an ApoTome (Zeiss).

To better examine cytoskeletal arrangement in salivary glands prior to histolysis, we used an anti-tubulin antibody to visualize the tubulin network (Fig. 4A–G). Control (*w^1118^*) and *sigmar^res^* strain salivary glands (at 25 hours APF) (Fig. 4A,B,D; supplementary material Fig. S3A) showed a continuous tubulin network. In contrast, the *sigmar^S^* null mutant salivary glands (at 25 hours APF) had a highly disrupted MT network pattern (Fig. 4C); in the grossly enlarged cells, very little tubulin was observed (Fig. 4C,E) but most of the normal sized cells showed a dense tubulin network with long fibrous MTs (Fig. 4F,G) that was not observed in the wild-type glands. Based on the tubulin phenotype we further categorized the MT network into normal (Fig. 4H), fragmented (Fig. 4I), sparse (Fig. 4J) and dense (Fig. 4K). While wild-type salivary gland cells at 23–24 hours APF harbored a normal (100%) tubulin network, *sigmar^S^* mutant salivary gland cells had normal (10.4%), fragmented (9.3%), sparse (71.8%), or dense (8.6%) tubulin networks (Fig. 4L). These observations indicate abnormal morphogenesis and a disrupted cytoskeletal arrangement in *sigmar^S^* mutant salivary glands.

### Sigmar is required for proper autophagic flux in death-stage salivary glands

**Fig. 5.**
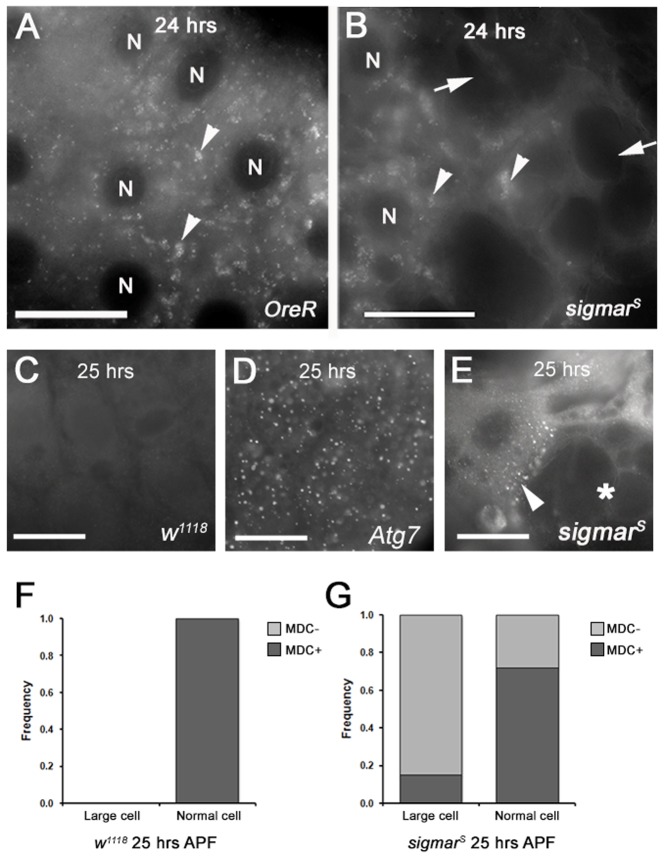
Salivary glands from *sigmar^S^* null mutants show defects in autophagy. (A) In control *OreR* salivary glands at 24 hours APF (at 18°C), monodansylcadaverine (MDC)-positive autolysosomes appear as punctate structures throughout the cells (arrowheads). (B) In the *sigmar^S^* mutant, some salivary gland cells show MDC-positive autolysosomes (left half of B, arrowheads). However, some enlarged cells show dramatically reduced MDC positive puncta (right half of B) and large vacuole-like structures (arrow). (C) In control *w^1118^* salivary glands, Ref(2)P protein aggregates were not detected. (D) In the *Atg7* mutant salivary glands, Ref(2)P protein aggregates were observed in all cells, indicative of a defect in autophagic flux. (E) In the *sigmar^S^* mutant salivary glands, some of the abnormal cells, typically adjacent to grossly enlarged/vacuolized cells (*), contained abundant Ref(2)P protein aggregates. At 24 hours APF, salivary glands (n = 16) from *w^1118^* (F) and *sigmar^S^* (G) pupae were dissected and the frequency of MDC+ and MDC- cells (corresponding to either normal sized or grossly enlarged cellular phenotypes; X axis) were quantified from a total of 159 cells and 155 cells per genotype, respectively, and the data is presented as a bar graph. Scale bars in A,B equal 250 microns, and in C,D,E equal 100 microns. Nuclei are marked with ‘N’ in A and B. Images were taken with a Zeiss Axioplan2 microscope.

Since MTs are required for the fusion of autophagosomes with endosomes and lysosomes (Blommaart et al., 1997; Köchl et al., 2006) to form autolysosomes, we determined whether autolysosomes were disrupted in the *sigmar^S^* mutant salivary glands. In a previous report (Akdemir et al., 2006) we showed that monodansylcadaverine (MDC), an acidotropic dye that detects acidic structures including autolysosomes, overlapped with the signal derived from GFP-LC3 (Rusten et al., 2004), a transgenic marker of autophagy, just prior to histolysis of *Drosophila* larval salivary glands at 24–26 hours APF (equivalent to 13–14.5 hours APF at 25°C). We thus analyzed MDC positive structures in salivary glands from *sigmar^S^* at 25–26 hours APF. In the wild-type salivary glands (Fig. 5A) and *sigmar^res^* strain (supplementary material Fig. S3B), MDC positive punctate structures appeared throughout the cells. In *sigmar^S^* null mutant glands, MDC positive structures appeared normal in some cells but were virtually absent in other cells (Fig. 5B). Thus, our findings suggest reduced autolysosome formation in at least some salivary gland cells in *sigmar^S^* null mutant animals. To determine the relative abundance of autolysosomes in normal sized and enlarged cells, we quantified the MDC positive cells in the wild-type and *sigmar^s^* salivary gland cells. Wild type salivary glands were composed entirely of normal sized cells that were 100% MDC positive. In *sigmar^S^* mutant salivary glands 50.6% of cells were normal sized and only 71.7% of these were MDC positive; the remaining 49.4% percent of cells were enlarged and only 14.8% of these were MDC positive.

To examine whether the reduced MDC-positive structures in some cells of dying larval salivary glands from *sigmar^S^* are associated with a decrease in autophagic flux, we examined salivary gland cells using the *Drosophila* Ref(2)P antibody (Nezis et al., 2008; Wyers et al., 1995) at early (16 hours APF) and late (25–26 hours APF) stages. Ref(2)P, a *Drosophila* homolog of the mammalian p62 protein, localizes to age-induced protein aggregates as well as to aggregates resulting from reduced autophagic or proteasomal activity (Nezis et al., 2008). Ref(2)P accumulates when autophagy is blocked, and thus Ref(2)P levels can be used as a readout for autophagic flux (degradative completion of autophagy). In control *w^1118^* salivary glands, we did not detect any Ref(2)P accumulation at either stage (Fig. 5C) as expected. As an added control, we examined the salivary glands of autophagy defective *Atg7* null-mutant animals (Juhász et al., 2007). We observed an accumulation of Ref(2)P protein in both early and late stages (Fig. 5D) which may be due to defects in both basal and death-stage autophagy, respectively. In *sigmar^S^* salivary glands some cells clearly showed Ref(2)P protein accumulation in the late death-stage. Ref(2)P protein accumulation was most evident in the cells that are adjacent to the grossly enlarged cells (Fig. 5E) and that typically contain an abnormally “dense” MT network (Fig. 4K). Our results suggest that autophagic flux is defective in *sigmar^S^* mutant late-stage salivary glands.

### Sigmar co-immunoprecipitates with the Ste20-like kinase Misshapen, and *sigmar^S^* mutants alter pDJNK localization in salivary glands

**Fig. 6.**
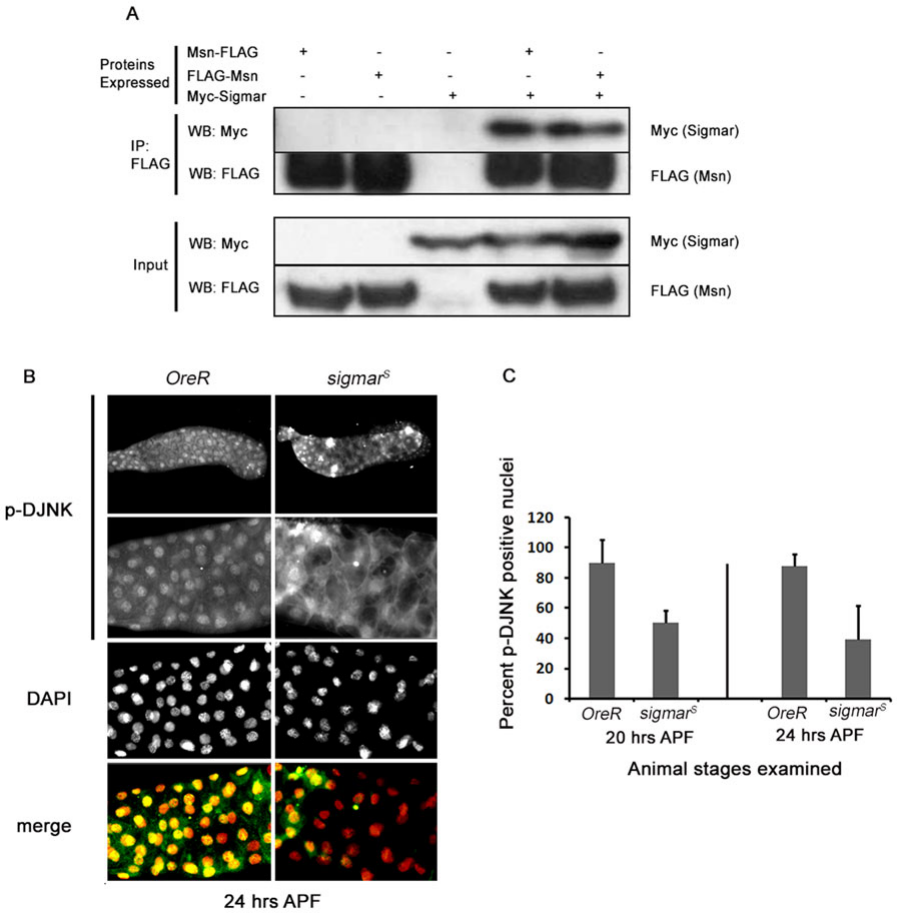
Sigmar is associated with the DJNK signaling pathway. (A) Sigmar interacts with Msn. S2 cells were co-transfected with C- or N-terminally FLAG-tagged Msn and Myc-Sigmar. Control cells were transfected with Myc-Sigmar only. Immunoprecipitations (IP) 72 hours after transfections with anti-FLAG antibody were performed and Sigmar was detected using anti-Myc antibody. The figure shown is representative of three independent IP replicates. (B) pDJNK localization is altered in *sigmar^S^* mutant salivary glands. *OreR* salivary gland at 24 hours APF shows pDJNK immunostaining in all nuclei (pDJNK = green, DAPI = red). The *sigmar^S^* salivary gland at 24 hours APF shows diffuse cytoplasmic staining of pDJNK in most cells although some regions (left) show nuclear pDJNK localization. Whole salivary gland images (top panel) were captured using a 10× objective and the zoomed images were taken with a 20× objective (Zeiss Axioplan 2). (C) Quantitation of nuclear-localized pDJNK in *OreR* and *sigmar^S^* salivary glands at two time points. At both 20 hours APF and 24 hours APF, *sigmar^S^* salivary glands show reduced nuclear localization of pDJNK. At least 10 salivary glands were examined from each genotype at each time point (P<0.003).

TNF-induced signal transduction pathways generally involve kinase cascades including JNK pathways (Wu, 2007). We and others observed an increase in Ste20-like kinase *Misshapen* (*Msn*) transcripts and protein prior to salivary gland cell death (Gorski et al., 2003; Martin and Baehrecke, 2004). We also identified unique peptides corresponding to Msn in our Sigmar IP, but not in the vector control, indicating that Msn may form protein complexes with Sigmar (Table 1). Since Msn and its mammalian homologs have been linked previously to cell shape changes and cytoskeletal alterations (Cobreros-Reguera et al., 2010; Hu et al., 2004), we chose to further investigate the potential interaction between Msn and Sigmar. First, to validate the protein interaction, we conducted reciprocal co-immunoprecipitation (Co-IP) western blot experiments. We used N- or C-terminus FLAG-tagged Msn protein as bait and captured Myc-Sigmar protein from co-transfected *Drosophila* S2 cells (Fig. 6A). Myc-Sigmar did not IP in the absence of bait protein. These results confirm that Msn and Sigmar interact with each other in *Drosophila* S2 cells.

Msn functions to regulate the *Drosophila* Jun amino-terminal kinase (DJNK), Basket, during dorsal closure, a process involving extensive cytoskeletal rearrangements and cell shape changes (Garlena et al., 2010; Harden, 2002; Su et al., 1998b; Su et al., 2000). The DJNK pathway is also known to be regulated by the Drosophila TNF ligand Eiger (Igaki et al., 2002; Moreno et al., 2002). Given the induction of Sigmar expression by Eiger, and the interaction between Msn and Sigmar, we postulated that Sigmar may also function to regulate the DJNK pathway in the context of salivary gland development. As a readout of JNK activation, we utilized an antibody that detects the phosphorylated or active form of DJNK/Basket (Davis, 2000). Control *OreR* salivary glands (20 and 24 hours APF) demonstrated a uniform nuclear localization of pDJNK (Fig. 6B) indicative of active JNK-mediated signal transduction. However, in *sigmar^S^* mutants, large patches of salivary gland tissue were devoid of pDJNK nuclear staining, but diffuse cytoplasmic pDJNK staining of low intensity was detected (Fig. 6B). Quantitation of nuclear localized pDJNK indicated a significant reduction in pDJNK positive nuclei in *sigmar^S^* mutant salivary glands compared to *OreR* at two different time points (20 hours and 24 hours APF) examined (Fig. 6C; p<0.003). Together, these findings indicate that Sigmar interacts with Msn and functions upstream of DJNK. While both Msn and DJNK activation have been implicated in cytoskeletal remodeling, future studies are required to determine the contribution of these molecules to the observed *sigmar^S^* phenotypes.

## Discussion

In this report, we describe the creation of a *Drosophila* genetic model to help characterize the *in vivo* functions of TIPE family members. Previous studies reported that the *Drosophila* TNFAIP8 homolog *sigmar* was transcriptionally upregulated prior to salivary gland histolysis, a developmental process associated with cytoskeletal rearrangement, apoptosis, and autophagy (Akdemir et al., 2006; Baehrecke, 2000; Baehrecke, 2003; Berry and Baehrecke, 2007; Gorski et al., 2003; Jiang et al., 1997; Jochová et al., 1997; Lee et al., 2000; Lee et al., 2003; Martin and Baehrecke, 2004; Yin and Thummel, 2005). Here we show that overexpression of Eiger, the *Drosophila* TNF-α-like ligand, can enhance sigmar expression levels in salivary glands. Eiger is also known to regulate the DJNK pathway and we found that Sigmar protein interacts with Msn, and *sigmar* loss-of-function leads to altered pDJNK distribution in salivary gland cells. *sigmar^S^* mutant salivary glands are morphologically abnormal with large empty vacuole-like structures, tubulin disorganization, and reduced autophagy flux. Together, these findings suggest similarities between *Drosophila* Sigmar and human TNFAIP8 regulation, and also provide new insights into cellular and molecular mechanisms associated with the function of this TIPE family member.

Our study is the first to implicate a TIPE family member in the regulation of JNK pathway signaling *in vivo*. While mammalian TNF-α signaling was associated previously with the regulation of JNK (Baud and Karin, 2001; Jupp et al., 2001; Liu et al., 1996) and more recently with TNFAIP8 (Horrevoets et al., 1999; Kumar et al., 2000) and TIPE2 (Sun et al., 2008), a connection between TIPE family function and JNK was not established. Several lines of evidence support a role for *Drosophila sigmar* in JNK signaling. First, the *Drosophila* JNK signaling pathway is activated in response to Eiger and we found that Sigmar is also upregulated by Eiger. Second, Sigmar protein interacts with Msn, a Ste20-like kinase that regulates DJNK. Lastly, in wild-type late-stage salivary glands, pDJNK is normally detected in the nuclei of salivary gland cells. However, in *sigmar^S^* mutant salivary glands, pDJNK was detected predominantly in the cytoplasm at low levels. Thus, our results indicate that Sigmar interacts with Msn, and functions downstream of Eiger and upstream of JNK nuclear translocation. Sigmar may be associated with JNK pathway signaling in additional developmental stages and tissues where it could play a redundant, minor or context-specific role as defined previously for other pathway components (Gonda et al., 2012; Mihaly et al., 2001). Our gene expression analyses (supplementary material Fig. S1) and FlyAtlas Anatomical Expression Data (Chintapalli et al., 2007) indicate *sigmar* is expressed in brain, mid-gut and ovary in addition to other tissues during multiple developmental stages from embryogenesis to the adult; future studies will be required to delineate *sigmar* functions and its relationship to DJNK signaling and other processes in these tissues and stages.

Our study is the first to associate autophagy defects with the loss-of-function of a TIPE family member *in vivo*. Consistent with our finding, a recent report showed regulation of autophagy by TNFAIP8 following oxidative stress in dopaminergic neurons (Ha et al., 2014). In contrast to apoptosis that results in dismantling of the cytoskeleton, the autophagy process depends on an intact cytoskeletal network. Intermediate filaments and microfilaments are required for the initial formation of autophagosomes (for a review, see Blommaart et al., 1997), and MTs are necessary for the fusion of autophagosomes with endosomes and lysosomes (Blommaart et al., 1997; Köchl et al., 2006). Treatment of starved Chinese Hamster Ovary (CHO) cells with vinblastine, or primary rat hepatocytes with vinblastine or nocodazole (two MT depolymerizing agents), inhibited fusion of autophagosomes to endosomes and lysosomes (Köchl et al., 2006; Munafó and Colombo, 2001), and resulted in abnormally large vacuolar structures. The authors suggested that the large vacuoles observed are late autophagosomes that have acquired acidifying H+-ATPase, and therefore stained with MDC. Thus, our *in vivo* findings are consistent with a requirement for an intact cytoskeletal network to effect the extensive death-stage autophagy flux, and suggest that the autophagy defects observed in the *sigmar* loss-of-function mutant may be a result of the cytoskeletal alterations that are evident at much earlier time points, though we can't rule out other mechanisms.

Our results support a role for *sigmar* in larval salivary gland cytoskeletal remodeling events. Loss of *sigmar* function resulted in morphologically abnormal salivary glands as early as 2 hours APF, a development stage when extensive cytoskeletal rearrangements are known to occur (Jochová et al., 1997). The *sigmar^S^* salivary glands appeared irregular with some enlarged bulging cells containing big empty vacuoles. This phenotype persisted until salivary gland histolysis that appeared to occur normally though was delayed. In wild-type *Drosophila*, the onset of salivary gland cell death was evident at 24 hours APF at 18°C, and between 26 and 28 hours APF the salivary glands condensed and degraded. An extensive rearrangement and depolymerization of tubulin has been observed at salivary gland death stages with tubulin visible on the surface of salivary glands but not detected in the cytoplasm (Martin and Baehrecke, 2004). Ectopic expression of the caspase inhibitor p35 or a dominant negative form of initiator caspase Dronc inhibited salivary gland cell death and reverted the localization of tubulin similar to the cytoplasmic distribution observed in early larval salivary glands (16 hours APF). However, p35 expression did not affect actin distribution. The late and abundant gene expression of s*igmar* in late-stage (23 hours APF) salivary glands is consistent with the rearrangement and/or depolymerization of cytoskeletal components such as actin and MTs that has been observed during the execution (26–28 hours APF) steps of cell death in salivary glands (Blommaart et al., 1997; Jochová et al., 1997; Martin and Baehrecke, 2004). In further support of such a role, and in agreement with the study by (Martin and Baehrecke, 2004), we found that only surface tubulin was evident in wild-type salivary glands at death stages, (Fig. 4A,D; supplementary material Fig. S5), while *sigmar^S^* mutant salivary glands at death stages showed tubulin in the cytoplasm of normal sized cells and reduced or absent tubulin staining throughout the enlarged cells (Fig. 4C, arrows; supplementary material Fig. S6, arrowheads). We did not detect any obvious differences in actin between wild type and mutants at death stages (data not shown). Salivary gland cell death requires activation of caspases (Martin and Baehrecke, 2004; Resnik-Docampo and de Celis, 2011) and proper autophagy (Berry and Baehrecke, 2007) function. In *sigmar^S^* mutants, salivary gland histolysis still occurred but was delayed, suggesting that Sigmar is just one of multiple factors involved in this process. While autophagy flux was decreased in large regions of salivary gland tissue, it was not completely inhibited. It may be that the activation of caspases combined with the remaining autophagy activity was sufficient to eventually promote the degradation of salivary gland remnants.

In summary, our *in vivo* evidence indicates that Sigmar, the *Drosophila* homolog of TNFAIP8, acts to modulate JNK signaling, cytoskeletal rearrangement, and autophagy in larval salivary glands. Future studies will be required to address the inter-relationship of these processes in the context of Sigmar function and the development of salivary glands and other tissues. The JNK pathway has well-established roles in tissue morphogenesis including cell shape changes, wound healing, cell migration and cell adhesion, and has also been linked to regulation of apoptosis and immunity (Davis, 2000; Stronach, 2005). A study in *Drosophila* (Wu et al., 2009) demonstrated a role for DJNK signaling in the transcriptional activation of several autophagy genes as well as induction of the autophagy process itself, in response to oxidative stress but not in response to developmental signals. During salivary gland histolysis, autophagy gene expression is regulated, at least in part, by ecdysone and the E93 transcription factor (Gorski et al., 2003; Lee et al., 2003), but we cannot rule out the possibility that Sigmar-mediated JNK signaling may also be involved. However, given the dependency of autophagy on an intact cytoskeleton, it is plausible that the autophagy alterations in the *sigmar^S^* loss-of-function mutant are secondary to the observed cytoskeletal disruptions though this will require further investigations. Either way, our study indicates that TIPE family member mutant phenotypes may also be related to alterations in autophagy, the dysregulation of which has been associated with inflammation, cancer, and the pathogenesis of multiple other human diseases. Further studies of *Drosophila* Sigmar may provide additional insights into the roles of TNFAIP8 and other TNFAIP8-like (TIPE) family members in normal development and in disease.

## Materials and Methods

### Probe preparation and salivary gland *in situ* hybridization

The larval salivary glands were dissected from pupae at 16 and 23 hours APF incubated at 18°C. Salivary glands were fixed immediately with 4% paraformaldehyde (PF) and subjected to whole-mount *in situ* hybridization with DNA probes. Gel purified PCR product was used to prepare single stranded anti-sense and sense DNA probes by a second round PCR with either Sigmar-43F40 or Sigmar-557R39 primers (supplementary material Table S1). After hybridization at 43°C overnight, the salivary glands were mounted in glycerol, viewed using a Zeiss Axioplan 2 microscope (Zeiss, NY, USA), and imaged with a cooled mono 12 bit camera (Qimaging, Surrey, Canada) and Northern Eclipse image analysis software (Empix Imaging Inc.).

### Quantitative RT-PCR

Salivary glands were dissected from animals at 16, 20, and 23 hours APF at 18°C, placed in Trizol (Life Technologies), homogenized, centrifuged at 12,000 ***g*** and stored at −80°C for up to two weeks. Trizol extractions of RNA were carried out as per the manufacturer's instructions (Life Technologies).

Primers (supplementary material Table S1) were designed using Primer Express V software (Applied Biosystems). Reactions were performed in triplicate using the SYBR Green One-step RT-PCR reagent kit on an Applied Biosystems 7900 Sequence Detection System as described elsewhere (Gorski et al., 2003). Reactions (15 µl) contained 50 ng of DNAse-treated (Life Technologies) total RNA and 0.1 µM of each primer. Melting curve analysis was performed for each run to ensure there was a single major product corresponding to the predicted melting temperature. Results were calculated using the Comparative CT Method (User Bulletin #2, ABI Prism 7700 Sequence Detection System, Applied Biosystems, 2001) with *Drosophila* rp49 as the reference gene; values were normalized to the 16 hours APF time point for salivary glands. *Drosophila* rp49 showed no significant difference in expression at the time points studied.

### Plasmid construction

Plasmids were constructed using the GATEWAY system (Life Technologies) as follows: A PCR product of the open reading frame (ORF) of *sigmar* was amplified from a full length cDNA construct of *sigmar* using primers containing *Att*B1 and *Att*B2 sequences. PCR products containing *Att*B1 and *Att*B2 sites were cloned into an entry clone, pDONR™221 (Life Technologies), containing *Att*P sites. The entry clones were sequenced to verify and confirm that the construct was in the correct orientation, in-frame and had no base pair change during PCR amplification. The entry clones were then used to shuttle the protein-coding region of *sigmar* into GATEWAY expression vectors containing either N-or C-terminal FLAG (*Drosophila* Genomics Resource Center). Expression of the correct size proteins was confirmed by western blotting. Sequences of the primers used for plasmid construction are provided in supplementary material Table S1.

### *Drosophila* cell culture and transfections

S2 cells (Life Technologies) were grown in ESF921 serum free (Expression systems) medium in 25 cm^2^ suspension flasks (Sarstedt) at 25°C. All experiments were carried out 3–4 days after passage and the cells were discarded after 25 passages. For transfection experiments, 3 µg of plasmid DNA and 10 µl of Cellfectin (Life Technologies) were combined in 200 µl of serum-free Grace medium (Life Technologies) for 30 min. Immediately prior to transfection, 3×10^6^ cells in 800 µl of Grace medium were prepared and incubated with the transfection medium (a total of 1 ml culture) overnight in a 24-well suspension culture plate (Sarstedt). Cells were equally split into two wells, and each well then received 1 ml of ESF921 serum-free medium. Cells were incubated for additional 24–48 hours before immunofluorescence experiments.

### *sigmar* loss of function construct

A fly strain containing a transposable p element in the 3′UTR region of the *sigmar* gene was identified and obtained from the Gene Disruption project (Bellen et al., 2004), but is now available from the Bloomington *Drosophila* Stock Center at Indiana University (*y^1^ w^67c23^; P(EPgy2)sigmar^EY06821^*). The female flies from this strain were crossed with males from strains of y^1^w^*^; CyO, H(PDelta2-3)HoP2.1/Bc^1^, which contain active transposase to excise the p element. Female progeny were crossed to male *yw; Gla/CyO* flies (a gift from Dr. N. Harden, Vancouver, Canada) and male progeny with white and glass (*Gla*) eyes or male or female progeny with white eye and curled wings (*CyO*), indicating excision of p elements, were established as stocks (sigmar^*^/*CyO*; here ^*^ indicates excision of the p element and potential deletion in the Sigmar region). A total of 538 stocks were established and a single fly from each stock was screened by genomic PCR using primers flanking the insertion site (Sigmar-1136R and l(2)dtl 2723R, supplementary material Table S1). This genomic PCR screen identified a deletion strain *sigmar^Df-C23^* (*Df(2R)l(2)dtl-sigmar/CyO; MKRS;TM6B*) whose PCR product was approximately 1.2 kb (wild-type band is approximately 6 kb). Both the *sigmar* and *l(2)dtl* coding regions were deleted in this strain and the *sigmar^Df-C23^/sigmar^Df-C23^* flies are lethal at the embryonic stage. This strain was rescued for the *l(2)dtl* gene by crossing it to several *pUAST-l(2)dtl* strains. One *pUAST-l(2)dtl^42-3^* strain rescued the *sigmar^Df-C23^* animals based on the observation that the progenies *sigmar^Df-C23^/sigmar^Df-C23^*; *pUAST-l(2)dtl^42-3^/pUAST-l(2)dtl^42-3^* and *Df-C23/Df-C23; pUAST-l(2)dtl^42-3^/TM6B* were viable. The *sigmar^Df-C23^/sigmar^Df-C23^; pUAST-l(2)dtl^42-3^/TM6B* strain was confirmed as a null mutant for *sigmar* by QRT-PCR analysis for the expression of *sigmar* and *l(2)dtl*. These mutants had no expression of *sigmar* but did express the *l(2)dtl* gene; this strain is referred to as *sigmar^S^*. To rescue the *sigmar* gene (ie. to create a control strain for loss of function studies) in the *sigmar^S^* strain, it was crossed to *pUAST-sigmar^15-1^/CyO-TM6B* flies and the progeny named as *sigmar^res^ (sigmar^Df-C23^/sigmar^Df-C23^; pUAST-l(2)dtl^42-3^/pUAST-sigmar^15-1^* (non-Tubby). QRT-PCR analysis confirmed the expression of *sigmar* and *l(2)dtl* in the *sigmar^res^* strain.

### Construction of *pUAST-sigmar* and *pUAST-l(2)dtl* strains

A full length *sigmar* cDNA clone constructed in pSPORT vector plasmid (Life Technologies) was digested with SalI and XbaI (New England Biolabs; NEB) and ligated into a pUAST vector (a gift from Dr. Tom Grigliatti, Vancouver, Canada) that was restriction digested with XhoI and XbaI (NEB). A full length cDNA clone of *l(2)dtl* in pOT2 was obtained from the *Drosophila* Genomics Resource Center and restriction digested with EcoRI and XhoI. The digested ORF region was ligated with a pUAST vector that was restriction digested with XhoI and EcoRI. Ligated constructs were transformed into DH10B-T1^R^ chemically competent cells (Life Technologies). Transformed cells were grown in 25 ml cultures and the plasmid DNA was prepared using HiPure plasmid purification kit (Life Technologies). Purified plasmid was combined with pTurbo helper plasmid (a gift from Dr. Tom Grigliatti, Vancouver, Canada) at 5:1 ratio to a total of 1 µg/µl (in 5 mM KCl, 0.1 mM NaPO_4_, pH 6.8) and ethanol precipitated. These plasmid DNA preparations were sent to the CBRC Transgenic *Drosophila* Fly Core facility (Massachusetts, USA) for injection into fly embryos. The transgenic flies with red eyes were identified by crossing the emergents with white eyed *w^1118^*. The red eye flies were established as stocks and the insert-containing chromosome was mapped by crossing with *w^1118^/Dp(1;Y)y^+^; CyO/nub^1^ b^1^ noc^Sco^ lt^1^ stw^3^*; *MKRS/TM6B*, *Tb^1^.* Initially, 72 strains for *l(2)dtl* and 60 strains for *sigmar* were generated. All the studies were conducted subsequently using the *pUAST-l(2)dtl^42-3^* strain for *l(2)dtl* gene and *pUAST-sigmar^15-1^* or the *pUAST-sigmar^29-9^* strain, which showed highest levels of expression by QRT-PCR (data not shown).

### Eiger overexpression in salivary glands

For salivary gland tissue-specific ectopic expression of Eiger, we crossed the *D59-SG-GAL4* strain (salivary gland II chromosome driver (Gustafson and Boulianne, 1996); kindly provided by Carl Thummel, Salt Lake City, USA) or *w^1118^; P(GawB)c729* (salivary gland III chromosome driver; Bloomington stock center) with *UAS-eiger* (Igaki et al., 2002) and analyzed the pupal salivary glands from the F1 progeny.

### MDC staining

Salivary glands were dissected at indicated time points in Schneider's medium (Life Technologies) or ESF921 serum-free medium and were incubated in the medium containing 1 µg/ml MDC for 30 minutes at 25°C. The salivary glands were washed once with medium, mounted in the medium and viewed using filter sets with 365 nm excitation and 397 nm emission in the Axioplan microscope. For quantification of MDC positive cells, images from 5–11 salivary glands per genotype were captured and at least 155 cells per genotype scored manually for the presence or absence of MDC positive puncta.

### Immunofluorescence (IF)

Approximately 75–100 µl of transfected S2 cells were placed into 8-well CC2 coated chamber slides (Nunc) and incubated for at least 30 min–4 hours or overnight as required. Cells were either fixed immediately with 4% paraformaldehyde for at least 20 minutes or received their respective treatment before fixing. For actin and microtubule disruption experiments, 10 µM latrunculin B (Calbiochem) for 5–25 minutes or 50 µM vinblastine sulfate salt (Sigma) for 35 minutes was employed, respectively. Fixed cells were washed several times with 1× Phosphate buffer saline (10× PBS, pH 7.4 from Life Technologies) and permeabilized with 0.2% Triton X100 for 5 min. A standard immunofluorescence protocol with primary and secondary antibody was followed thereafter. Cells were either incubated for 3 hours at room temperature or overnight at 4°C with primary anti-FLAG mouse (Sigma), anti-FLAG rabbit (Sigma), or anti-β-tubulin (Hybridoma bank) antibodies in a humidified chamber. Incubation with the secondary antibodies anti-mouse Alexa 488 (Life Technologies), anti-mouse CY3 (Jackson's laboratory), anti-rabbit Alexa 488 (Life Technologies) or anti-rabbit CY3 (Jackson's laboratory) was carried out at room temperature for two hours. For phalloidin staining, cells were incubated for 15 minutes in a 1:250 dilution (from 6.6 µM stock) of phalloidin-rhodamine (Life Technologies) just before mounting. After incubations with antibodies, cells were mounted in Slowfade mount (Life Technologies).

Salivary glands were dissected into 4% paraformaldehyde and fixed for at least 30 minutes at room temperature. Salivary glands were then permeabilized with 1% Triton 100, pre-incubated in 1× PBS (Life Technologies) containing 0.2% Triton and 1% BSA for 1 hour at room temperature. Salivary glands were then incubated in anti-β-tubulin mouse antibody (E7; Hybridoma bank) at a 1:100 dilution or in anti-Ref(2)P rabbit polyclonal antibody at a 1:500 dilution (Nezis et al., 2008; Wyers et al., 1995) or in anti-pDJNK (Cell Signaling mouse antibody at a 1:200 dilution in PBS containing 0.2% Triton and 1% BSA overnight at 4°C in a humidified chamber. A secondary anti-mouse CY3 antibody (Jackson laboratory) or anti-mouse Alexa 488 (Life Technologies) anti-rabbit Alexa 546 (Life Technologies) was used to detect tubulin and Ref(2)P proteins respectively. Mounting were followed as described above for the S2 cell IF.

### Confocal or ApoTome image acquisition and analysis

Tissues and cells were viewed with a Nikon C1 confocal microscope. S2 cell images were obtained using a Plan Apochromat 60×/1.45 NA oil immersion objective (Nikon). Salivary gland images were obtained using 20×/0.75 NA objective lens. Images were captured and analyzed with EZ-C1 Ver 3.00 software. Alternatively, tissues and cells were viewed using an Axioplan 2 microscope (Zeiss, NY, USA) with ApoTome (Zeiss) and imaged with a Zeiss AxioCam MRm. Images were captured and analyzed using AxioVision software. For S2 cell images and salivary gland images, a 64× lens and 20× lenses were used respectively. For quantification of tubulin network phenotypes, 10 salivary glands each from wild type and mutant were analyzed, with two images/gland captured using the ApoTome and Zeiss AxioCam MRm camera. Cells displaying normal, fragmented, sparse and dense tubulin networks were manually counted. For Z-stack series, images were captured using 40× lenses at 2 µm thickness.

### Immunoprecipitation (IP) and tandem mass spectrometry

The large scale IP experiment was repeated a total of 7 times with N- or C-terminus FLAG-tagged Sigmar protein as baits. For each experiment, 90 ml of the transfected culture per expression construct was pelleted and then resuspended in 10 ml lysis buffer (20 mM Tris pH 7.5, 150 mM NaCl, 1 mM EDTA, NP-40 (0.1–5%), 10 mM β-glycerophosphate, 2 mM Sodium orthovanadate, 10 µg/ml leupeptin, 2 µg/ml aprotinin, 1 mM AEBSF, 10 µg/ml pepstatin A, 10 µg/ml Leupeptin, 10 µg/ml Aprotinin). Cell extract was clarified by centrifugation and passing through 0.15 µm nylon filter, followed by pre-clearing with 50% slurry of Sepharose 4B (Sigma-Aldrich). Supernatant was then incubated with 40 µl of 50% anti-FLAG M2 agarose (Sigma-Aldrich) for 3 hours or overnight at 4°C to capture interaction partners. FLAG-tagged protein complexes were eluted with FLAG peptide (Sigma-Aldrich). Eluates were vacuum dried and resuspended in protein sample buffer (1×MES buffer+1×protein loading buffer, Life Technologies) and were separated by SDS-PAGE on a 10–20% NuPAGE gradient gel (Life Technologies). Protein bands visualized by colloidal Coomassie stain were extracted, reduced and S-alkylated as described (Shevchenko et al., 1996; Wilm et al., 1996), followed by in-gel trypsin digestion overnight at 37°C. Resulting peptides were extracted under basic or acidic (50% v/v acetonitrile, 5% v/v formic acid) conditions. Peptide mixtures were subjected to LC-MS/MS analysis on a Finnigan LCQ (PTRL West) or 4000QTRAP (Applied Biosystems) ion trap mass spectrometers via reversed phase HPLC nano-electrospray ionization. All MS/MS spectra were queried against *Drosophila* Ensembl release sequence databases (Flicek et al., 2014) using Mascot (Matrix Sciences, London, UK) or X!tandem algorithms (Craig and Beavis, 2004). Protein hits were considered reliable when 2 or more unique peptides were assigned to high quality (at least e-value <−3) or when 1 unique peptide with e-value ≤−2 was observed in conjunction with at least 2 peptides in another experiment. An in-house web-based database called SpecterWeb was developed to store and process raw mass spectrometric protein identifications (Mead et al., 2010). SpecterWeb software subtracted proteins found in vector-only controls from those identified in the cells transfected with Sigmar bait constructs. The results were shown in Table 1 and supplementary material Table S2.

For reciprocal co-expressed Co-IP experiments, S2 cells were transfected with FLAG-Msn (or Msn-FLAG) and/or Sigmar-Myc, and after 72 hours, 40 ml of transfected S2 cells were pelleted and then lysed in 1 ml lysis buffer and processed as described above. For western blots, samples with 1×NuPAGE LDS Buffer (Life Sciences) were run on NuPAGE Bis-Tris gels (10%, Life Technologies). Membranes were blocked in 2% skim milk and incubated with anti-FLAG antibody (rabbit, Sigma) and anti-Myc antibody (mouse, Roche) overnight (4°C) followed by HRP-conjugated 2° antibodies (Santa Cruz Biotechnology) and detection with ECL reagent (Amersham) or SuperSignal substrate (Thermo Scientific).

### Histology

Flies were reared and maintained at 21°C. *w^1118^* and *sigmar^s^* (*sigmar ^Df-C23^/sigmar ^Df-C23^); pUAST-l(2)dtl^42-3^/TM6B*) animals were aged to 24 hours APF (18°C), fixed in FAAG (80% ethanol, 4% formaldehyde, 5% acetic acid, 1% glutaraldehyde) for 16 hours at 4°C as previously described (Berry and Baehrecke, 2007), paraffin embedded, sectioned and stained with hemotoxylin and eosin (H&E). Sections were imaged on a Zeiss Axiophot II microscope.

### Statistical analyses

As indicated in the legends, statistical significance was calculated by Student's *t* test when comparing differences between two samples. P-value<0.05 was considered statistically significant.

### Abbreviations

SG, salivary gland; CT, cycle threshold; PCD, programmed cell death; APF, After Puparium Formation; QRT-PCR, quantitative reverse transcription PCR; MT, microtubule.

## Supplementary Material

Supplementary Material
