# Supplementary material for: The Drosophila TIPE family member Sigmar interacts with the Ste20-like kinase Misshapen and modulates JNK signaling, cytoskeletal remodeling and autophagy
Source: Biol Open. 2015 Apr 2;4(5):672–84. doi: 10.1242/bio.20148417 (PMC4434819; doi:10.1242/bio.20148417)
Supplement: Supplementary Material [file supp_4_5_672__index.html]

The Drosophila TIPE family member Sigmar interacts with the Ste20-like kinase Misshapen and modulates JNK signaling, cytoskeletal remodeling and autophagy — The Drosophila TIPE family member Sigmar interacts with the Ste20-like kinase Misshapen and modulates JNK signaling, cytoskeletal remodeling and autophagy — Supplementary Material 

# The *Drosophila* TIPE family member Sigmar interacts with the Ste20-like kinase Misshapen and modulates JNK signaling, cytoskeletal remodeling and autophagy

## bio.20148417 Supplementary Material

**Files in this Data Supplement:**

- Supplementary Material - Suganthi Chittaranjan et al. doi: 10.1242/bio.20148417
- Table S2 - Candidate interaction partners of Sigmar
