# Supplementary material for: The Drosophila TIPE family member Sigmar interacts with the Ste20-like kinase Misshapen and modulates JNK signaling, cytoskeletal remodeling and autophagy
Source: Biol Open. 2015 Apr 2;4(5):672–84. doi: 10.1242/bio.20148417 (PMC4434819; doi:10.1242/bio.20148417)
Supplement: Supplementary Material [file supp_bio.20148417_bio.20148417-s1.pdf]

Biology Open (2015) 000, 1–13 doi:10.1242/bio.20148417

Supplementary Material

Suganthi Chittaranjan et al. doi: 10.1242/bio.20148417

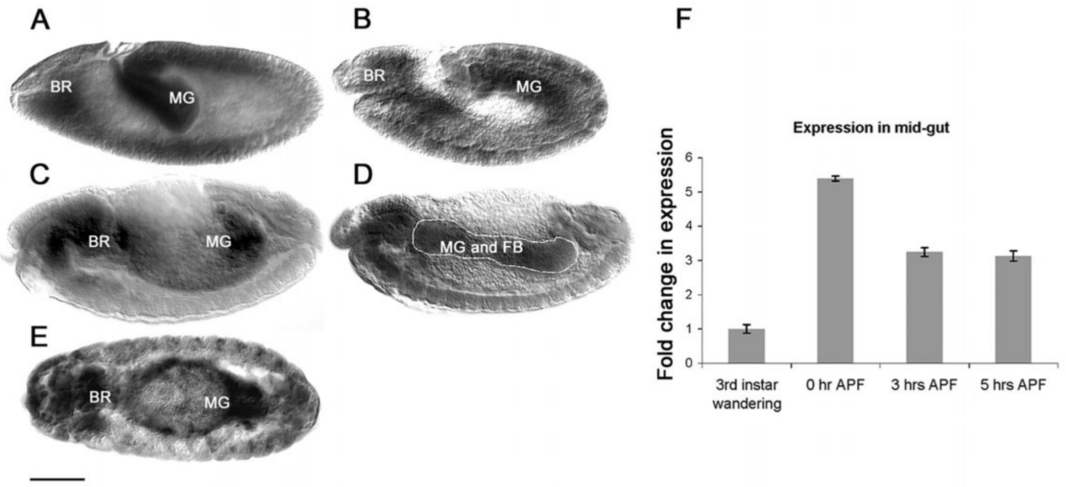

Fig. S1. ***sigmar*** **is expressed in** ***Drosophila*** **embryonic brain and midgut, and is increased in midgut at puparium formation.** (A,B) *In-situ* hybridization of *Drosophila* embryos at stage 9–10 shows strong expression of *sigmar* in the midgut (MG) and brain (BR). Lateral view. (C,D) Drosophila embryos at late stage 13–16 show expression of *sigmar* in the brain (BR), midgut (MG) endoderm and its precursors, as well as fat body (FB) (D; outlined areas). Lateral view. (E) Dorsal view of *Drosophila* stage 13 embryo shows *sigmar* expression in brain (BR) and midgut (MG). (F) qRT-PCR analysis indicates that *sigmar* expression in larval midgut (mean±s.d.) is increased at 0 hr APF, after the ecdysone pulse. At 3 hours and 5 hours APF, *sigmar* expression was slightly reduced but remained elevated as compared to the level in 3rd instar wandering larvae. Scale bar in A–E represent 100 microns.

Biology Open

Biology Open (2015) 000, 1–13 doi:10.1242/bio.20148417

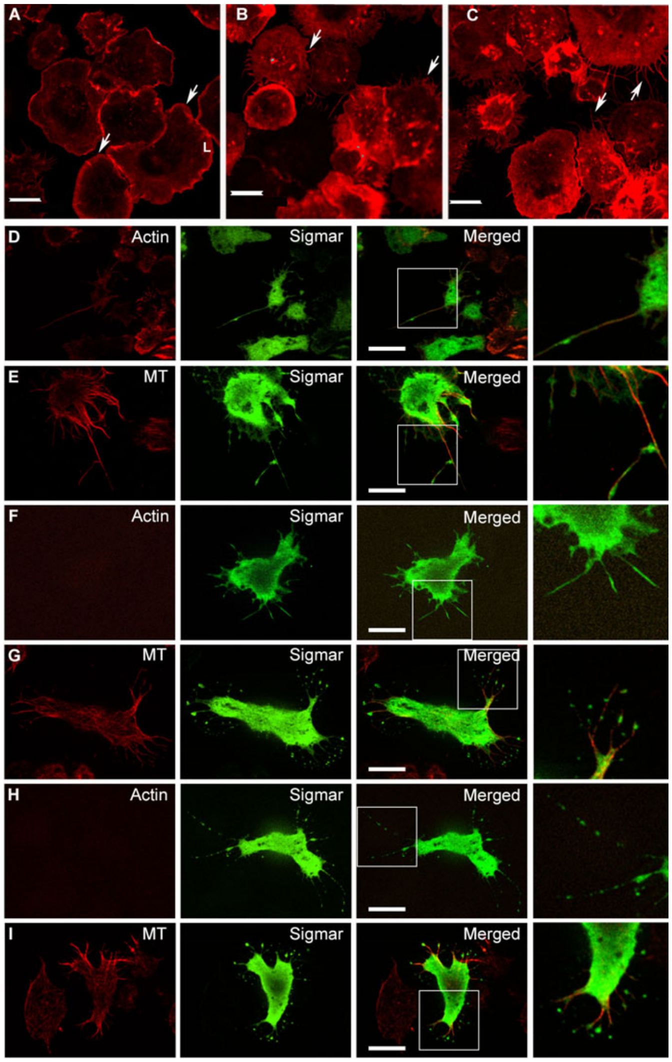

Fig. S2. ***Drosophila*** **S2 cells grown for more than 3 hours on CC2 coated slides attach, flatten, spread and form short and long extensions, to which Sigmar localizes irrespective of latrunculin treatment.** S2 cells were transfected with plasmid, grown on CC2 coated slides for 30 min, 3 hours or 5 hours, fixed with 4% paraformaldehyde and stained for actin with rhodamine-phalloidin. After 30 minutes (A) cells attached themselves to the slides, flattened, spread and formed lamellae. The cells appeared symmetrical and extensive actin staining was observed in the lamellae 'L' area (arrows). This morphology remained for up to 1 hour (data not shown). After 3 hours, at least 50% of the cells showed short protrusions or long extensions that stained positive for actin (B, arrows). After 4–5 hours, most of the cells had long extensions around the periphery (C; arrows). Scale bars in images A–C are 10 microns. Images were captured using a Nikon confocal microscope and shown is a single Z slice. Myc-Sigmar transfected S2 cells were allowed to attach on CC2 coated slides for 24 hours and treated with 10 µM latrunculin for 5 (D,E), 15 (F,G) or 25 (H,I) minutes. Cells were then fixed with 4% paraformaldehyde and stained with anti-Myc antibody and either rhodamine-phalloidin (Actin) or anti-tubulin antibody (MT). Actin network was partially intact at 5 minutes (D) and absent at 15 minutes (F) and 25 minutes (H). Tubulin network was not affected by latrunculin treatment (E,G and I) and Sigmar can be seen localizing to protrusions regardless of latrunculin treatment (E–I). Boxed areas show the regions magnified in subsequent panels. Scale bars in D–I represent 10 microns.

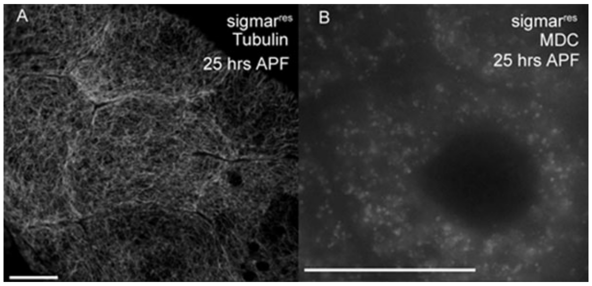

Fig. S3. Salivary glands of ***sigmar^{res}*** **animals are similar to wild-type glands.** (A) Confocal image of salivary gland from the *sigmar* and *l(2)dtl* rescue strain (*sigmar^{res}*) at 25 hours APF show a normal surface tubulin network detected with anti-beta tubulin antibody. At least 70% of the salivary glands from the control *sigmar^{res}* strain appeared morphologically normal. Scale bar equals 20 microns. (B) Fluorescent microscopy image of a salivary gland from the same strain at 25 hours APF stained with MDC. Scale bar equals 50 microns.

Biology Open

Biology Open (2015) 000, 1–13 doi:10.1242/bio.20148417

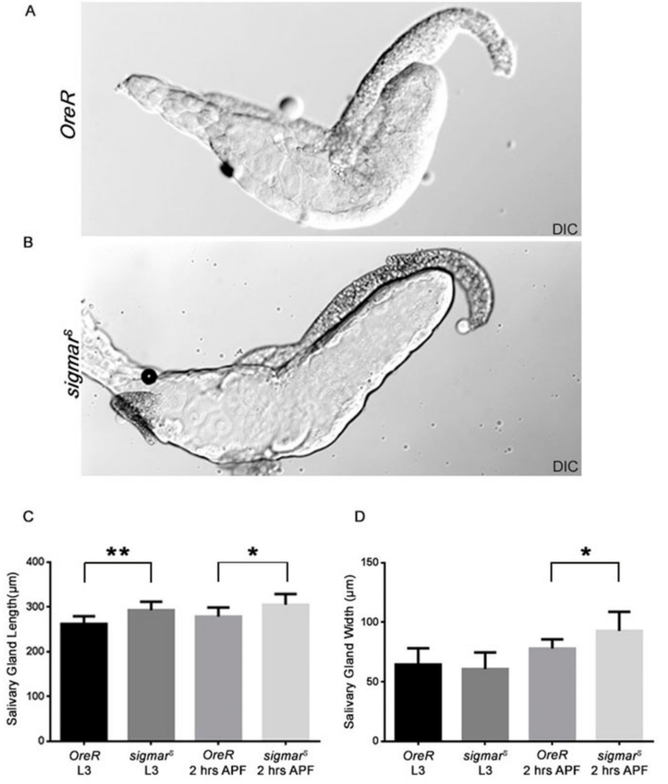

Fig. S4. Salivary glands of 3rd instar *sigmar^S* wandering larvae are morphologically similar to control glands. Differential interference contrast (DIC) images of salivary glands from (A) control (*OreR*) and (B) *sigmar^S* null 3rd instar wandering larvae. Though greater in length (p=0.002), morphology of *sigmar^S* salivary gland was comparable to that of the control at L3. Quantitation of (C) salivary gland length and (D) width in *OreR* and *sigmar^S* at L3 and 2 hours APF (mean±s.d.) indicates that *sigmar^S* glands were significantly longer (p=0.032) and wider (p=0.034) than their control counterparts at 2 hours APF, corresponding to the appearance of grossly enlarged cells. Images were captured with a Zeiss Axioplan 2 microscope (×10 objective). 8–10 salivary glands were examined for each genotype at each time point. Student's *t* test was used to compare differences between genotypes. * significant at $p<0.05$; ** significant at $p<0.01$.

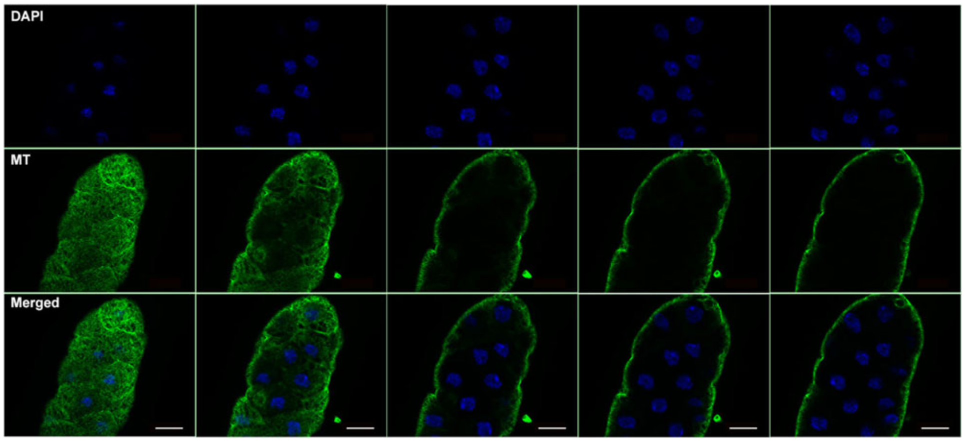

Fig. S5. Z-stack image of 23 hours APF control salivary gland. At 23 hours APF, *w^{1118}* salivary glands display an intact and evenly distributed tubulin network detectable only on the outer gland surface. (A) Nuclear DAPI staining (blue), (B) anti-tubulin antibody staining (green) and (C) overlay are shown for a representative gland from outer surface toward interior (left to right panels). All cells are comparable in shape and size. Z-stack images were captured using 40× objective with ApoTome at 2 μm step size. Scale bars represent 20 microns.

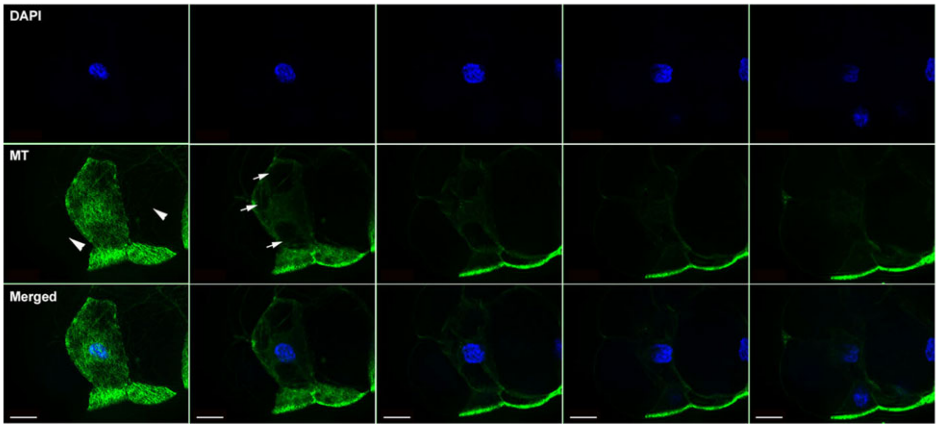

Fig. S6. Z-stack image of 23 hours APF *sigmar*^{S} salivary gland. At 23 hours APF, *sigmar*^{S} salivary glands appear abnormal and enlarged overall. (A) Nuclear DAPI staining (blue), (B) anti-tubulin antibody staining (green) and (C) overlay are shown from a representative gland from outer surface toward interior (left to right panels). Some cells are grossly enlarged compared to those of the control, and are devoid of tubulin staining (arrowheads). In other cells, the tubulin network extends beneath the gland outer surface and into the cytoplasm. Vacuole-like structures surrounded by microtubules can sometimes be seen in the cytoplasm (arrows). Z-stack images were captured using 40× objective with ApoTome at 2 µm step size. Scale bars represent 20 microns.

Table S1. Sequences of primers used in this study

Primer name	Sequence (5′–3′)
Eiger-277F_20	GCATCCTCAGCCTCAAATGA
Eiger-428R_22	CCTGAAGCTCTGTGTGATTTCC
l(2)dtl 2723R	GCCGGCACACTGCTACTTCT
Rp49-68F	AGTCGGATCGATATGCTAAGCT
Rp49-165R	AGATACTGTCCCTTGAAGCGG
Sigmar-229F	CGGAACAATCAGTTCAGCGA
Sigmar-319R	GATATGATGGACAGCTGCGTG
Sigmar-1136R	AAGGTTACCGACCCCTTGGA
Sigmar-43F40	TAATACGACTCACTATAGGGCGCAGAAGAAGATCCTCTCA
Sigmar-557R39	AATTAACCCTCACTAAAGGTTCCATGGCTGCGTTTATGT

Table S2: See supplementary webpage
