# Supplementary material for: The Drosophila TIPE family member Sigmar interacts with the Ste20-like kinase Misshapen and modulates JNK signaling, cytoskeletal remodeling and autophagy
Source: Biol Open. 2015 Apr 2;4(5):672–84. doi: 10.1242/bio.20148417 (PMC4434819; doi:10.1242/bio.20148417)
Supplement: Supplementary Material [file supp_bio.20148417_Table_S2.docx]

| **Table S2. Candidate interaction partners of Sigmar**  Additional proteins that immunoprecipitated with Sigmar, but not the empty vector control, are listed here. Column 1 indicates the FlyBase Annotation symbol for polypeptide of the interacting proteins and Column 2 indicates the Gene Symbol. Molecular mass of each polypeptide is indicated in the third column. Column 4 indicates the molecular function as described in FlyBase. A total of seven immunoprecipitation experiments (IP), 4 experiments with N-terminus and 3 experiments with C-terminus Sigmar-FLAG fusion proteins (column 5) were performed and the protein peptides identified in both C-terminus and N-terminus IPs are shown. The number of times each protein peptide identified is shown in column 6. The log(E) value in column 7 is an estimate of the probability that the protein assignment occurred randomly as calculated by the X!Tandem algorithm using the IPI (International Protein Index) protein database. The number of unique peptides contributing to the log(E) value is shown in column 8. | | | | | | | | | | |
| --- | --- | --- | --- | --- | --- | --- | --- | --- | --- | --- |
|  | **Annotation symbol of polypeptide** | **Symbol** | **Mass (KDa)** | **Molecular function** | **N or C terminus 3XFLAG** | | **No. of times observed** | | **log(E)** | **Unique** |
| 1 | **Sigmar-PA** | **sigmar** | 21.4 | involved in autophagic cell death | N-FLAG | | 4/4 | | -151.3 | 18 |
|  |  |  |  |  |  | |  | | -130.3 | 14 |
|  |  |  |  |  |  | |  | | -98 | 12 |
|  |  |  |  |  |  | |  | | -54.9 | 9 |
|  |  |  |  |  | C-FLAG | | 3/3 | | -90.3 | 11 |
|  |  |  |  |  |  | |  | | -58.4 | 9 |
|  |  |  |  |  |  | |  | | -64.1 | 8 |
|  | **Lipid metabolism associated** | | | | | | | | | |
| 2 | CG4581-PA | thiolase | 50.6 | acetyl-CoA C-acyltransferase activity; long-chain-3-hydroxyacyl-CoA dehydrogenase activity | | N-FLAG | | 3/4 | -47.9 | 9 |
|  |  |  |  |  | |  | |  | -25.6 | 4 |
|  |  |  |  |  | |  | |  | -2.8 | 2 |
|  |  |  |  |  | | C-FLAG | | 2/3 | -51.9 | 8 |
|  |  |  |  |  | |  | |  | -1.3 | 2 |
| 3 | CG18212-PB | alt1 | 95 | unknown function; found in lipid droplet fractions from Drosophila embryos Cermelli et. al. 2006 | | N-FLAG | | 2/4 | -56.9 | 7 |
|  |  |  |  |  | |  | |  | -46 | 6 |
|  |  |  |  |  | | C-FLAG | | 1/3 | -45.9 | 7 |
| 4 | CG11198-PA |  | 278.4 | acetyl-CoA carboxylase activity; ATP binding | | N-FLAG | | 2/4 | -22.2 | 5 |
|  |  |  |  |  | |  | |  | -13.5 | 3 |
|  |  |  |  |  | | C-FLAG | | 1/4 | -19.4 | 3 |
|  | **DNA binding and nucleogenesis** | | | | | | | | | |
| 5 | CG10223-PA | Top2 | 164.3 | DNA binding; DNA topoisomerase (ATP-hydrolyzing) activity; mRNA binding | | N-FLAG | | 1/4 | -81.2 | 20 |
|  |  |  |  |  | | C-FLAG | | 1/3 | -90.1 | 19 |
| 6 | CG7421-PB | Nopp140 | 70.5 | involved in nucleologenesis | | N-FLAG | | 2/4 | -142.2 | 16 |
|  |  |  |  |  | |  | |  | -49.2 | 1 |
|  |  |  |  |  | | C-FLAG | | 2/3 | -22.6 | 8 |
|  |  |  |  |  | |  | |  | -31.9 | 1 |
| 7 | CG7421-PA |  | 72.1 |  | | N-FLAG | | 3/4 | -62.4 | 9 |
|  |  |  |  |  | |  | |  | -8.7 | 2 |
|  |  |  |  |  | |  | |  | -104.6 | 1 |
|  |  |  |  |  | | C-FLAG | | 2/3 | -31.9 | 7 |
|  |  |  |  |  | |  | |  | -16 | 1 |
| 8 | CG6143-PB | Pep | 78 | single-stranded DNA binding | | N-FLAG | | 2/4 | -40 | 7 |
|  |  |  |  |  | |  | |  | -42.7 | 6 |
|  |  |  |  |  | | C-FLAG | | 1/3 | -48.6 | 5 |
| 9 | CG8545-PA |  | 99.8 | nucleic acid binding. | | N-FLAG | | 1/4 | -13 | 6 |
|  |  |  |  |  | | C-FLAG | | 1/3 | -2.4 | 3 |
| 10 | CG3178-PA | Rrp1 | 74.6 | DNA binding; DNA-(apurinic or apyrimidinic site) lyase activity; double-stranded DNA 3'-5' exodeoxyribonuclease activity | | N-FLAG | | 1/4 | -16.8 | 6 |
|  |  |  |  |  | | C-FLAG | | 1/3 | -7.7 | 3 |
| 11 | CG3231-PA | snama | 138.9 | nucleic acid binding; ubiquitin-protein ligase activity; zinc ion binding | | C-FLAG | | 1/3 | -2.3 | 3 |
|  |  |  |  |  | | N-FLAG | | 1/4 | -1.2 | 2 |
| 12 | CG6061-PA | mip120 | 99.9 | DNA binding; protein binding | | N-FLAG | | 2/4 | -9.2 | 2 |
|  |  |  |  |  | |  | |  | -2.7 | 1 |
| 13 | CG10712-PB | Chro | 101 | chromatin binding | | N-FLAG | | 2/4 | -11 | 2 |
|  |  |  |  |  | | C-FLAG | | 1/3 | -9.2 | 2 |
|  | **Other functions** | | | | | | | | | |
| 14 | CG6226-PA | FK506-bp1 | 39.3 | FK506 binding; peptidyl-prolyl cis-trans isomerase activity | | C-FLAG | | 1/3 | -37.5 | 10 |
|  |  |  |  |  | | N-FLAG | | 2/4 | -22.1 | 5 |
|  |  |  |  |  | |  | |  | -3.9 | 1 |
| 15 | CG3074-PB | Swim | 48.7 | Wnt-protein binding; cysteine-type endopeptidase activity; polysaccharide binding; scavenger receptor activity | | N-FLAG | | 3/4 | -44.7 | 9 |
|  |  |  |  |  | |  | |  | -33.8 | 5 |
| 16 | CG8715-PA | lig | 149.4 | ubiquitin-associated protein | | N-FLAG | | 1/4 | -46.7 | 6 |
|  |  |  |  |  | | C-FLAG | | 1/3 | -7.7 | 2 |
| 17 | CG4145-PC | Cg25C | 174.2 | extracellular matrix structural constituent; structural molecule activity | | N-FLAG | | 3/4 | -20 | 4 |
|  |  |  |  |  | |  | |  | -9 | 2 |
|  |  |  |  |  | |  | |  | -8 | 2 |
| 18 | CG1994-PA | l(1)G0020 | 112.8 | N-acetyltransferase activity | | N-FLAG | | 2/4 | -27.4 | 4 |
|  |  |  |  |  | |  | |  | -3.5 | 1 |
|  |  |  |  |  | | C-FLAG | | 1/3 | -3.7 | 2 |
| 19 | CG9281-PB |  | 69.4 | ATP binding; ATPase activity, coupled to transmembrane movement of substances; transporter activity | | N-FLAG | | 2/4 | -8.5 | 4 |
|  |  |  |  |  | | C-FLAG | | 1/3 | -11.5 | 2 |
| 20 | CG10686-PA | tral | 69.3 | protein binding | | C-FLAG | | 1/3 | -7.1 | 4 |
|  |  |  |  |  | | N-FLAG | | 1/4 | -6.6 | 1 |
| 21 | CG5214-PA |  | 49.9 | dihydrolipoyllysine-residue succinyltransferase activity; acetyltransferase activity | | N-FLAG | | 2/4 | -12.6 | 4 |
|  |  |  |  |  | |  | |  | -3.5 | 1 |
| 22 | CG6375-PB | pit | 76.9 | ATP-dependent RNA helicase activity; | | C-FLAG | | 1/3 | -13.2 | 3 |
|  |  |  |  |  | | N-FLAG | | 2/4 | -10.9 | 3 |
|  |  |  |  |  | |  | |  | -2.7 | 1 |
| 23 | CG43758-PA | sli | 165.8 | receptor binding; calcium ion binding | | N-FLAG | | 1/4 | -4.4 | 3 |
|  |  |  |  |  | | C-FLAG | | 1/3 | -1.9 | 3 |
| 24 | CG5519-PA | Prp19 | 55.1 | ubiquitin-protein ligase activity | | C-FLAG | | 1/3 | -7.9 | 3 |
|  |  |  |  |  | | N-FLAG | | 1/4 | -12.7 | 2 |
| 25 | CG1685-PA | pen | 81.2 | involved in apposition of dorsal and ventral imaginal disc-derived wing surfaces | | N-FLAG | | 2/4 | -17.2 | 2 |
|  |  |  |  |  | | C-FLAG | | 1/3 | -4 | 2 |
|  | **Predicted functions** | | | | | | | | | |
| 26 | CG1677-PA |  | 109 | nucleic acid binding; zinc ion binding | | N-FLAG | | 3/4 | -92.7 | 10 |
|  |  |  |  |  | |  | |  | -27.8 | 4 |
|  |  |  |  |  | |  | |  | -10.4 | 4 |
|  |  |  |  |  | | C-FLAG | | 1/3 | -18.2 | 5 |
| 27 | CG18178-PA |  | 42.5 | Nucleotide-binding | | N-FLAG | | 1/4 | -19.6 | 5 |
|  |  |  |  |  | | C-FLAG | | 1/3 | -2.1 | 1 |
| 28 | CG13096-PA |  | 74.3 | RNA binding | | C-FLAG | | 1/3 | -8.9 | 4 |
|  |  |  |  |  | | N-FLAG | | 2/4 | -13.5 | 3 |
|  |  |  |  |  | |  | |  | -2.8 | 1 |
| 29 | CG5516-PA |  | 18.9 | nucleic acid binding | | C-FLAG | | 1/3 | -12.7 | 2 |
|  |  |  |  |  | | N-FLAG | | 1/4 | -10 | 2 |
|  | **Unknown function** | | | | | | | | | |
| 30 | CG2691-PA |  | 153.4 | unknown function | | N-FLAG | | 2/4 | -18.7 | 4 |
|  |  |  |  |  | |  | |  | -22.1 | 3 |
|  |  |  |  |  | | C-FLAG | | 2/3 | -7.3 | 1 |
|  |  |  |  |  | |  | |  | -2.5 | 1 |
| 31 | CG12909-PA |  | 32.1 | unknown function | | N-FLAG | | 2/4 | -16.8 | 3 |
|  |  |  |  |  | |  | |  | -1.6 | 3 |
|  |  |  |  |  | | C-FLAG | | 1/3 | -5.9 | 1 |
| 32 | CG12792-PA | l(2)09851 | 50.6 | unknown function | | C-FLAG | | 1/3 | -9.8 | 2 |
|  |  |  |  |  | | N-FLAG | | 2/4 | -3.9 | 1 |
|  |  |  |  |  | |  | |  | -2.9 | 1 |
| 33 | CG15784-PA |  | 62.3 | unknown function | | N-FLAG | | 1/4 | -11.8 | 2 |
|  |  |  |  |  | | C-FLAG | | 1/3 | -7.4 | 1 |
